# Mitochondrial Small Heat Shock Proteins Are Essential for Normal Growth of *Arabidopsis thaliana*

**DOI:** 10.3389/fpls.2021.600426

**Published:** 2021-02-10

**Authors:** Mariela R. Escobar, Ivo Feussner, Estela M. Valle

**Affiliations:** ^1^Instituto de Biología Molecular y Celular de Rosario (IBR-CONICET-UNR), Rosario, Argentina; ^2^Department of Plant Biochemistry, Albrecht-von-Haller-Institute for Plant Sciences, Göttingen Center for Molecular Biosciences (GZMB), University of Göttingen, Göttingen, Germany

**Keywords:** growth arrest, heat stress, metabolism, proteomics, sHSP

## Abstract

Mitochondria play important roles in the plant stress responses and the detoxification of the reactive oxygen species generated in the electron transport chain. Expression of genes encoding stress-related proteins such as the mitochondrial small heat shock proteins (M-sHSP) is upregulated in response to different abiotic stresses. In *Arabidopsis thaliana*, three *M-sHSPs* paralogous genes were identified, although their function under physiological conditions remains elusive. The aim of this work is to uncover the *in vivo* function of all three *M-sHSPs* at the whole plant level. To accomplish this goal, we analyzed the phenotype, proteomic, and metabolic profiles of Arabidopsis knock-down lines of *M-sHSPs* (single, double, and triple knock-down lines) during normal plant growth. The triple knock-down plants showed the most prominent altered phenotype at vegetative and reproductive stages without any externally applied stress. They displayed chlorotic leaves, growth arrest, and low seed production. Concomitantly, they exhibited increased levels of sugars, proline, and citric, malic, and ascorbic acid, among other metabolites. In contrast, single and double knock-down plants displayed a few changes in their phenotype. A redundant function among the three M-sHSPs is indicated by the impairment in vegetative and reproductive growth associated with the simultaneous loss of all three *M-sHSPs* genes. The triple knock-down lines showed alteration of proteins mainly involved in photosynthesis and antioxidant defense compared to the control plants. On the other hand, heat stress triggered a distinct cytosolic response pattern and the upregulation of other sHSP members, in the knock-down plants. Overall, depletion of all three M-sHSPs in Arabidopsis severely impacted fundamental metabolic processes, leading to alterations in the correct plant growth and development. These findings expand our knowledge about the contribution of organelle-specific M-sHSPs to healthy plant growth under non-stress conditions.

## Introduction

In plants, reactions involved in regular metabolisms, such as photosynthesis and respiration, are sources of reactive oxygen species (ROS) in the cell as the unavoidable consequence of aerobic life (Halliwell and Gutteridge, 2015). Under heat stress, mitochondria generate much more ROS than other cell compartments (Zhang et al., 2009). To cope with heat stress, plants produce small heat shock proteins (sHSPs) as the most dominant proteins (Sun et al., 2002), representing the first lines of cellular defense against irreversible protein aggregation that arise during various stresses (Waters, 2013). sHSPs are also known to act in the acquired heat stress tolerance (Song et al., 2012).

Plant sHSPs respond to a wide range of environmental stresses (Wang et al., 2004; Jacob et al., 2017), and *sHSP* gene expression is mediated by the increase in cellular H_2_O_2_ accumulation in *Arabidopsis thaliana* (Vanderauwera et al., 2005; Scarpeci et al., 2008a). Nevertheless, *sHSP* genes are hardly expressed in vegetative tissue under non-stress conditions (Waters et al., 1996; Scarpeci et al., 2008b).

The protective function of sHSPs in stress responses is quite conserved among different plant species. Overexpression of tea (*Camellia sinensis*) *sHSP* genes confers tolerance to heat and cold stress in Arabidopsis (Wang et al., 2017). Also, sHSP21 is responsible for the development of chloroplasts during heat stress (Zhong et al., 2013).

The presence of organelle-targeted sHSPs appears to be unique to plants (Waters, 2013; Waters and Vierling, 2020), except for a mitochondrion-targeted sHSP found in *Drosophila melanogaster* (Morrow and Tanguay, 2015) and the parasite *Toxoplasma gondii* (de Miguel et al., 2005).

In Arabidopsis, two homologous genes encoding mitochondrial (M-) sHSPs, M-sHSP23.5 (*At5g51440*), and M-sHSP23.6 (*At4g25200*) (Scharf et al., 2001; Hooper et al., 2017) and a later identified gene (*At1g52560*) encoding an M-sHSP26.5, also of mitochondrial location (Siddique et al., 2008; Hooper et al., 2017), were found to be very highly co-expressed during stress conditions in more than 150 microarray experiments (Leister et al., 2011). In particular, *At5g51440* was rapidly and highly upregulated in Arabidopsis seedlings by superoxide anions (O_2_^⋅_–_^) generated in the chloroplast during photosynthesis by the herbicide methyl viologen (Scarpeci et al., 2008a) and by application of 100 mM hydrogen peroxide (Hieno et al., 2019). It was recently reported that one of the six genes encoding cotton (*Gossypium hirsutum*) M-sHSP accelerates seed germination in response to increased temperature via ROS generation (Ma et al., 2019). The authors reported that the cotton M-sHSP could bind to cytochrome C maturation protein, blocking the cytochrome C in the mitochondrial electron transport chain. Previously, it was demonstrated that *D. melanogaster* DmHsp22 improves maximal mitochondrial oxygen consumption capacity and ATP content, providing a mechanistic link between DmHsp22 and mitochondrial functions (Dabbaghizadeh et al., 2018).

The function of M-sHSP in thermotolerance has been previously studied, focusing on the overexpression of *sHSPs* in different plants (Jiang et al., 2009). Transgenic plants were produced using orthologous *M*-*sHSPs* genes of Arabidopsis, maize (*Zea mays*), or tomato (*Solanum lycopersicum*). Tomato “Micro-Tom” plants transformed for high expression of *M-sHSP23.6* from Arabidopsis participate in the heat tolerance mechanism, while the suppression of this protein resulted in more significant physiological damage during heat stress (Huther et al., 2013). Arabidopsis plants expressing a transgene encoding the maize *ZmHSP22* showed increased thermotolerance and altered expression of nuclear genes encoding the endogenous M-sHSP23.6 and several HSPs, suggesting that the heat-induced mitochondrial retrograde regulation was altered (Rhoads et al., 2005). Tobacco (*Nicotiana tabacum*) plants expressing an *M−sHSP* gene from tomato exhibited thermotolerance, whereas the antisense plants in which the expression of the gene is suppressed exhibited susceptibility to heat stress (Sanmiya et al., 2004).

The set of stress-resistant phenotypes described above for *M-sHSP* overexpressing transgenic material suggests the importance of M-sHSPs for the plant to survive at high temperatures, although it is essential to recognize the limited nature of the heat tolerance demonstrated up to now. As proposed, sHSPs function as ATP-independent chaperones that capture stress-denaturing proteins to prevent irreversible denaturation (Waters and Vierling, 2020). Currently, there is a lack of knowledge about the *in vivo* function of sHSPs, particularly the organelle-specific sHSPs, during the plant growth cycle.

In this work, we aim to demonstrate the importance of each M-sHSP at the whole plant level. Accordingly, we generated Arabidopsis knock-down of all the three genes [single, double (*At4g25200*, *At5g51440*), and triple knock-down lines] using artificial microRNA (amiR) strategy, and proteomic and metabolomic approaches. Evidence is provided for the critical role of the three M-sHSPs in the overall cell homeostasis.

## Materials and Methods

### Plant Materials and Growth Conditions

The *A. thaliana* Col-0 ecotype was used in all the experiments. The plants were grown in a growth chamber at 22°C ± 2°C under 16 h light/8 h dark with fluorescent light at 120 μmol m^–2^ s^–1^ and 60% humidity.

### Generation of Artificial microRNA Silenced Plants

The generation of knock-down Arabidopsis was performed by using a constitutive amiR approach, according to Schwab et al. (2006). For the single knock-down *amiR* lines, optimal amiR sequences to knock down the genes *At5g51440*, *At4g25200*, and *At1g52560* were obtained from the WMD—Web Micro RNA designer tool^1^ and the miRNA^∗^ was designed to match the amiR in the same way as in the duplex miR–miR^∗^ of miRNA319 (Supplementary Table 1). Similarly, a specific amiR sequence for both *At5g51440* and *At4g25200* transcript was obtained for the double knock-down *amiR23.5/23.6* lines (*amiRD*). NB147 plasmid containing the MIR319a precursor was used to engineer the amiRs before being subcloned into the binary vector pCHF3 under the control of the 35S promoter. The same amiR sequences designed for the double and *amiR26.5* lines were in frame cloned under the control of a unique 355 promoter in the pCHF3 vector to obtain the triple *amiR23.5/23.6/26.5* construct. Stable transformation of Arabidopsis plants was performed by flower dip with *Agrobacterium tumefaciens* (Clough and Bent, 1998), and the presence of the transgene was confirmed by PCR. A minimum of four independent transforming lines was isolated for each construct, including the Col-0 (empty vector controls).

### Construction of Promoter–GUS Fusion Lines and GUS Staining

The 5′ sequence upstream of the translation initiation codon of the *At5g51440* (446 bp), *At4g25200* (1087 bp), and *At1g52560* (1100 bp) genes was amplified by PCR using specific primers (Supplementary Table 1) and cloned in-frame fused to the GUS reporter gene (1812 bp) contained into the pCHF3 binary vector. The constructs were used to transform Arabidopsis plants by floral dip. Five independent lines were isolated for each promoter construct. GUS staining was performed as described (Weigel and Glazebrook, 2002) under normal or heat stress conditions (3 h at 37°C). The staining was repeated in at least 20 biological replicates of 7- and 15-day-old plants from every independent line.

### Isolation of RNA From Tissues Plants

RNA from 7-day seedlings was isolated using TriPure Isolation Reagent (Sigma-Aldrich Co., St. Louis, United States) and following the manufacturer’s procedure. RNA concentrations were measured by using a NanoDrop 2000 spectrophotometer, and the quality was confirmed by loading 1 μg of the sample in a 1.5% (w/v) agarose gel. In all experiments, extractions were performed in four biological replicates of control or transgenic independent line. Twenty plants were pooled for one replicate.

### Quantitative RT-PCR (RT-qPCR)

Contaminant DNA was digested and removed from RNA suspension by using DNase I following the manufacturer’s instructions. The RNA was then reverse transcribed into cDNA with the RevertAid transcriptase. RT-qPCR measurements were performed by using the Takyon No Rox SYBR Core Kit blue dTTP following the manufacturer’s instructions and the iQ5 qPCR cycler. Gene expression was normalized to the protein phosphatase 2A gene (*PP2A*) that was used as a reference gene. Four biological replicates were measured in each control and transgenic independent line, and two technical replicates were performed.

### Root Length and Seed Germination Experiments

Sterilized Arabidopsis seeds were spread on petri dishes containing half-strength Murashige and Skoog medium and 0.8% (w/v) agar. After stratification for 3 days at 4°C in the dark, seeds were grown vertically under normal conditions, and the roots were photographed every day and measured for 15 days. Fourteen independent triple knock-down lines (*amiRT*) were evaluated, as well as three independent lines in the rest of the construct and controls. Eight biological replicates in each line were measured. For seed germination, approximately 80 sterilized seeds of each *amiR* line (four independent lines) and control lines were used. Nine days after stratification, the number of germinated seeds under normal conditions was counted.

### Pigment Content

Twenty milligrams fresh weight of plant material was extracted two times with 80% ethanol (Merck)/10 mM MES (pH 5.9) (Sigma-Aldrich) and once with 50% Ethanol/10 mM MES (pH 5.9) for 30 min at 80°C; the supernatants were combined and used for pigment content determination in a polystyrene 96-deep well plate as in Scarpeci et al. (2017). Four independent lines of each *amiR* construct, and eight biological replicates in every control and transgenic independent line were measured.

### Pulse Amplitude Modulation (PAM) Analyses

PSII photochemical response was evaluated through PAM fluorometric measurements by using a Dual-PAM-100 system (Heinz Walz, Effeltrich, Germany). Four independent lines and three biological replicates per line were measured for each *amiR* construct, as well as 12 control plants. Replicates consisted of individual plants. The experiment was repeated twice. Twenty-one-day-old control and *amiR* plants were used and dark adapted for 2 h before measurements. This allowed the complete inactivation of the Calvin-Benson cycle. A short induction protocol of 70 s was applied to the leaves consisting of illumination with actinic light of 166 μmol photons m^–2^ s ^–1^ and saturating pulses every 5 s. The information of photosynthetic parameters was extracted from the Dual-PAM-100 software as described in Klughammer and Schreiber (2008). The parameters Fv/Fm, ΦPSII, qL, ETR (II), NPQ, ΦNPQ, and ΦNO were evaluated during the 70 s of induction.

### ROS and Cell Death Detection

H_2_O_2_ and O_2_^⋅_–_^ were detected in 28-day-old plants as described in Scarpeci et al. (2008a). For O_2_^⋅_–_^ detection, leaves were vacuum-infiltrated with 50 mM sodium phosphate, pH 7.5, containing 0.2% (w/v) nitroblue tetrazolium (NBT) (N6876, Sigma-Aldrich) and incubated at room temperature for 2 h in the dark. For the detection of H_2_O_2_, leaves were infiltrated with a solution of 1 mg/ml 3,3′-diaminobenzidine (DAB). Trypan blue staining was performed following a modified protocol (Fernández-Bautista et al., 2016). Leaves were incubated in trypan blue staining solution (85% lactic acid 10 ml, phenol 10 ml, 99% glycerol 10 ml, distilled water 10 ml, and trypan blue 40 mg) for maximum 1 h. After all incubations, tissues were cleared with 70% ethanol before being photographed. Experiments were conducted in four independent lines of each *amiR* construct; eight biological replicates were measured in control and transgenic lines.

### Cell Areas Determination

Leaves from 21-day-old plants were incubated with lactic acid (85%) at room temperature for 3 days, until tissues were completely cleared. Leaf cells were detected using differential interference contrast microscopy in an Olympus BH2 microscope. To determine the mean areas, six leaves and a minimum of 650 epidermal and parenchymal cells from each knock-down line or control plants were measured.

### Electrolyte Leakage Measurement

Discs from 28-day-old Arabidopsis leaves were thoroughly rinsed with deionized water, covered with 0.4 M mannitol, and incubated at 25°C for 3 h. A conductance meter (Twin Compact Meter-Horiba, Northampton, United Kingdom) was used to measure the conductivity at 25°C after the incubation and again after autoclaving samples for 30 min at 120°C to release all the electrolytes. Electrolyte leakage was indicated as a percentage of total electrolytes. Three independent lines of each *amiR* and three biological replicates in each control and transgenic independent line were measured.

### Metabolite Profiling by GC-MS

Metabolites were extracted from 100 mg of ground leaf material of 15-day-old Arabidopsis plants. According to an established GC-MS protocol (Lisec et al., 2006), extractions were done by using a methanol/chloroform protocol followed by samples derivatization and measurement of the metabolite levels. Metabolites were identified by comparing to databases of authentic standards (Schauer et al., 2005) and quantified based on the internal standard added. Values were expressed as fold changes of metabolites in each sample relative to control plants. Experiments were performed in three biological replicates of control and *amiR* lines; replicates consisted of a pool of 50 plants.

### Proteomic Experiments

Proteins were isolated from 15-day-old Arabidopsis plants grown in half-strength Murashige and Skoog medium by using a phenol extraction protocol, according to Grimplet et al. (2009). Protein concentration was determined by using the protein quantitation assay kit BSA Thermo Scientific Pierce. Sixty micrograms of protein was loaded on a 10% SDS polyacrylamide gel and run until samples had migrated 1 cm into the running gel. The bands stained with Coomassie Brilliant Blue G-250 (Neuhoff et al., 1988) were excised from the gels and subjected to an in-gel tryptic digest as in Shevchenko et al. (2007). The peptides were analyzed with LC-combined tandem MS in the Service Unit LCMS Protein analytics at the University of Göttingen by the use of OrbitrapTM mass spectrometers coupled to UltiMate3000 RSLCnano systems. Data are available via ProteomeXchange with identifier PXD019603. Measurements were performed in three biological replicates of empty vector control and *amiR* lines; each replicate corresponds to one transgenic independent line and consisted of a pool of 50 plants. Two technical replicates were performed and combined in the data analysis.

### Proteomic Data Analysis

Protein groups were identified and quantified by performing label-free quantification (LFQ) with the MaxQuant software 1.6.2.10 (Cox and Mann, 2008) following standard procedure. The Arabidopsis Proteome ID UP000078284 database downloaded from UniProt was used. The resulting protein groups file was loaded into Perseus 1.6.1.1 (Tyanova et al., 2016), and the matrix was filtered using default settings. Technical replicates were combined in a new matrix. The data were transformed into log_2_ for the statistical tests and imputation. For the hierarchical clustering, the data were filtered to have at least three valid values in one group. Missing values were imputed from a normal distribution (witch: 3, down shifted: 1.8). Samples in one group were combined and the average was calculated. *Z* score normalization was applied and a hierarchical cluster with Euclidean distance, average linkage, and maximum 30 clusters was performed. Protein abundance changes were discovered by performing pair-based comparisons of the LFQ intensities between control and each *amiR* group using two-side *t*-test analysis. For this, the LFQ matrix was filtered to have three valid values in at least one of the groups in each comparison. Proteins identified with at least two unique peptides and statistically significant changes (*t*-test, *P* < 0.05) were considered for further analysis. Unless otherwise stated, relative LFQ (rLFQ) was calculated as a percentage of the total LFQ in the sample and was used to show the relative abundance of proteins in the proteome.

### Bioinformatics Analysis

Protein sequences were aligned using the Clustal2.1 program (EMBL-EBI), the percentage of identity was determined, and the CLC Sequence Viewer 7.0.2 was used to represent the conservation at a particular position in the alignment. The overrepresented Gene Ontology (GO) in the list of proteins was investigated by using STRING (Szklarczyk et al., 2019), and the subcellular localization was determined by SUBA 4 (Hooper et al., 2017). Venn diagrams were drawn on the Online Venn Diagram Tool.

### Statistical Analyses

Statistical significance in the pair-based comparisons was estimated according to Student’s *t*-test by using Excel 2016. Only comparisons with a *P*-value < 0.05 were designated as statistically significant. Proteomic data were statistically analyzed by Perseus 1.6.1.1 and Excel 2016 software.

## Results

### All Three *M-sHSP* Genes Are Heat-Inducible

**FIGURE 1:**
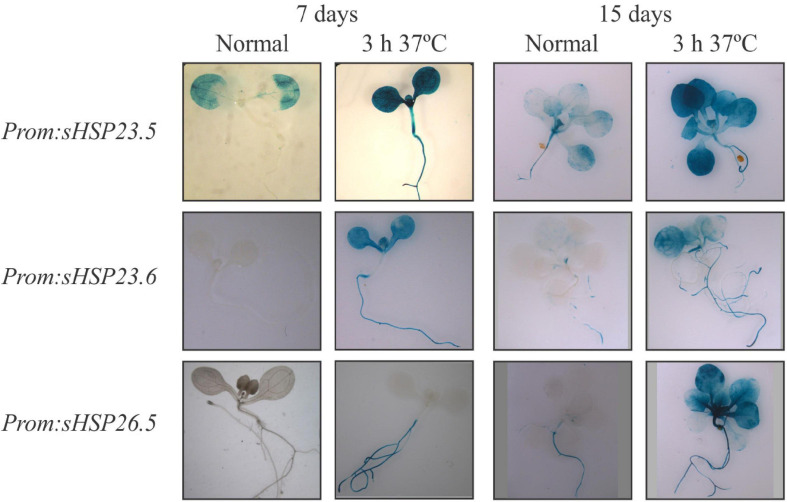
Histochemical analysis of *M-sHSP*s promoter activity in Arabidopsis seedlings. Transgenic plants carrying the promoter regions of *At5g51440* (−446 bp), *At4g25200* (−1087 bp), and *At1g52560* (−1100 bp) fused to the *GUS* gene were grown for 7 or 15 days under normal condition (22°C) and then exposed for 3 h to a higher temperature (37°C). GUS staining was performed as stated in Materials and Methods. Five independent transformed lines and a minimum of 20 biological replicates per line were tested for each construct, stage, and condition. Experiments were performed twice. Representative pictures of plants before and after stress treatments are shown.

The protein sequence alignment of the M-sHSP23.5, M-sHSP23.6, and M-sHSP26.5 revealed that the three mitochondrial proteins share high sequence similarity (Supplementary Figure 1A). The highest sequence conservation is observed between sHSP23.5 and sHSP23.6 (68% identity) (Supplementary Figure 1B), reflecting that *At5g51440* and *At4g25200* may be paralogous genes and the product of a duplication event (Waters et al., 2008). The heat response of *M-sHSPs* was assayed by RT-qPCR in 7-day-old Arabidopsis seedlings (whole seedling) exposed to 37°C for 3 h. Compared to the expression under normal growth conditions, the three *M-sHSP* genes were significantly and highly upregulated by heat (Supplementary Figure 2). To further evaluate this heat stress response in different tissues, the promoter region of *At5g51440* (446 bp), *At4g25200* (1087 bp), and *At1g52560* (1100 bp) was fused to the reporter gene *GUS*, and transgenic Arabidopsis seedlings were analyzed under normal growth conditions and heat stress (3 h at 37°C) (Figure 1). All three promoters were clearly responsive to heat stress and almost inactive under normal growth conditions in 7-day-old seedlings (Figure 1). At this stage, *M-sHSP23.5* and *M-sHSP23.6* promoters were active in all tissues after heat treatment, while *M-sHSP26.5* promoter activity was only detectable in the root. In 15-day-old seedlings, *M-sHSP23.5*, *M-sHSP23.6*, and *M-sHSP26.5* promoters were responsive to heat conditions in all tissues (Figure 1).

### The *M-sHSP amiR* Plants Show Differences in the Vegetative Phenotype

**FIGURE 2:**
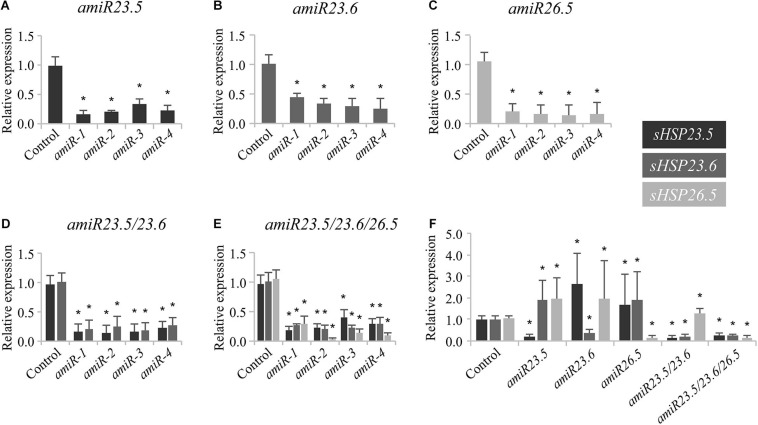
Expression of *sHSP23.5*, *sHSP23.6*, and *sHSP26.5* in knock-down *amiR* lines. Seedlings of *amiR23.5* **(A)**, *amiR23.6* **(B)**, *amiR26.5* **(C)**, *amiRD* **(D)**, and *amiRT* **(E)** were grown for 7 days at 22°C under long-day conditions and then exposed 3 h to 37°C for expression induction, before sampling. All expression values are normalized to the protein phosphatase 2A gene (*PP2A*) as a reference. Relative expression of the transcripts was normalized to their respective expression in control plants. In each construct, four independent transgenic lines were evaluated for the expression of the silenced gene. Experiments were performed twice; data shown correspond to one representative experiment. Each data point represents the mean value ± SD of four biological replicates in every independent line. Twenty plants were pooled for one replicate. One of these four replicates of each independent line in one construct was selected to measure the expression of the three genes (the amiR target and the others). These four samples were taken as representative of the four independent lines and combined in the last panel **(F)**. Asterisks indicate significance by a two-sided *t*-test with *P < 0.05.

**FIGURE 3:**
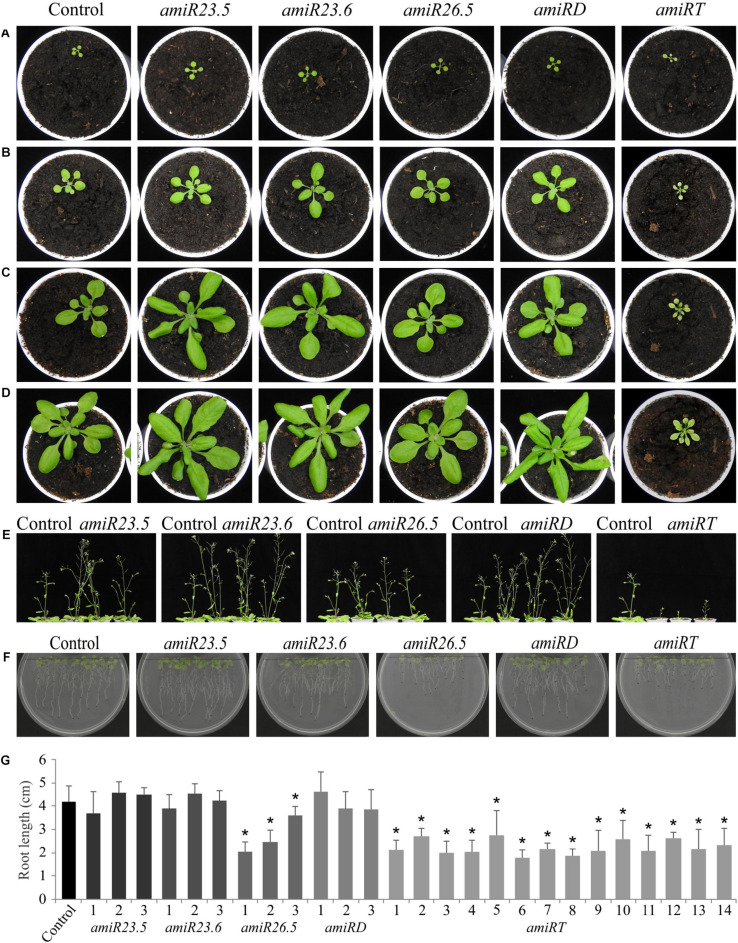
Phenotypes and root length of *amiR* plants. At least three independent transgenic lines of *amiR* constructs and control plants at different developmental stages were analyzed. Representative pictures of 15-day-old **(A)**, 20-day-old **(B)**, 25-day-old **(C)**, 28-day-old **(D)**, and 40-day-old **(E)** plants are shown. For the root length evaluation **(F,G)**, plants were vertically grown, and roots were periodically photographed and measured. Pictures **(F)** and values **(G)** correspond to the root length after 15 days of growth. Three independent lines of each *amiR* construct were evaluated, except for the *amiRT*, where 14 independent lines were evaluated **(G)**. Data points represent the mean value ± SD of eight biological replicates. Asterisks indicate significance by a two-sided *t*-test with *P < 0.01.

To assess the function of the M-sHSPs, we knocked down the three *M-sHSPs* in transgenic Arabidopsis plants by using a constitutive amiR approach. Since transfer DNA insertion lines (knockout) were not available for all of the individual genes, to avoid problems due to potential redundancy, and in order to make them comparable to the double and triple lines, knock-down transgenic plants were in parallel obtained for each of the *M-sHSP* by using an identical artificial microRNA strategy (Supplementary Figure 3). Transgenic plants were accordingly named as *amiR23.5*, *amiR23.6*, and *amiR26.5* for every single construct, *amiR23.5/23.6* (or *amiRD*) for the double knock-down, and *amiR23.5/23.6/26.5* (or *amiRT*) for the triple knock-down line. The posttranscriptional silencing of the target genes was confirmed by RT-qPCR in at least four independent transgenic lines obtained for each *amiR* construct (Figure 2). The single and *amiRD* lines, with downregulation of one or two of *M-sHSP*, responded with increased *M-sHSPs* expression of the others, indicating a transcriptional regulation and probable functional compensation of these proteins (Figure 2F). The phenotype of at least three independent transgenic lines of all *amiR* construct and empty vector control plants at different developmental stages was analyzed. Seven-day-old seedlings (not shown) and 15-day-old plants from *amiR* lines were equal in size to those of the control plants (Figure 3A). Soon after, 20-day-old plants of the single and double knock-down lines of *sHSP23.5* and *sHSP23.6* produced bigger rosettes than control plants (Figure 3B). Representative 25-day-old rosettes (Figure 3C) and 28- and 40-day-old plants (Figures 3D,E) show the apparent difference in the size of these plants. The single *amiR26.5* lines, on the other hand, developed a control-like phenotype at this stage (Figure 3E). The most extreme phenotype was found in the *amiR23.5/23.6/26.5*, where the complete development was affected (Figures 3B–E). These plants showed tiny plants in the whole growth cycle. They were significantly smaller, only half-height of the control plants, with apparently fewer branches than control plants, and the leaves were chlorotic. Although *amiRT* plants developed fully expanded rosettes, they develop an entirely different phenotype from all other transgenic lines (Figure 3E).

Concerning the roots, the phenotype and length in *amiR23.5*, *amiR23.6*, and *amiR23.5/23.6* lines were similar to control plants (Figures 3F,G). Differently, *amiR26.5* and *amiR23.5/23.6/26.5* plants produced shorter roots than control in all the evaluated lines (Figures 3F,G).

**FIGURE 4:**
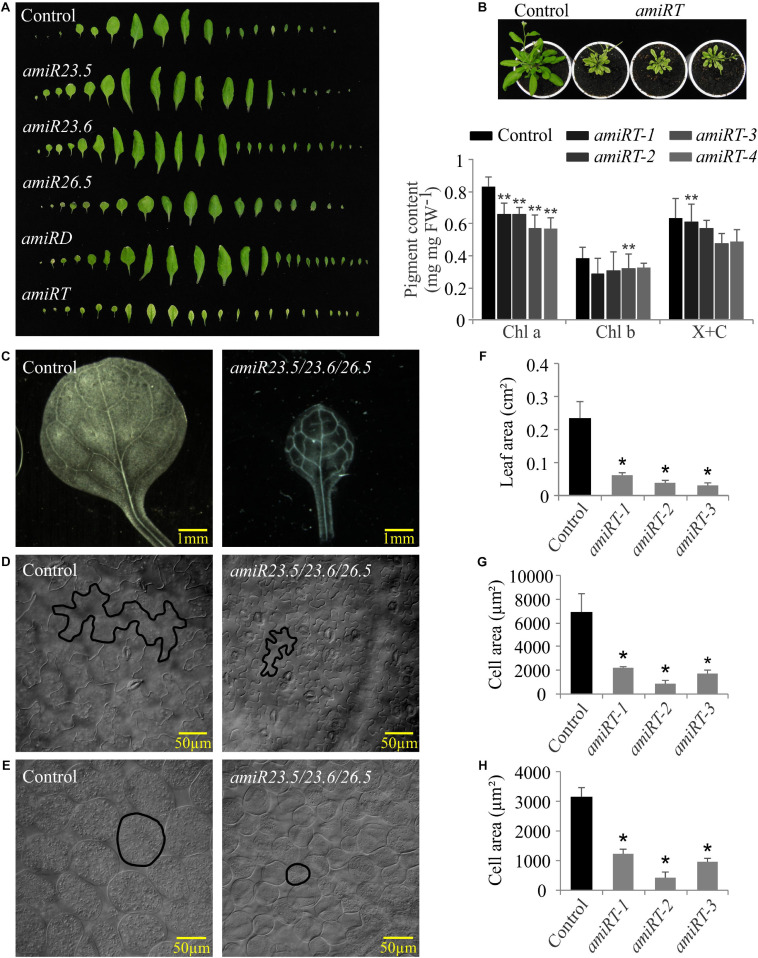
Leaf phenotypes of *amiR* plants. **(A)** Leaf series of the *amiR* knock-down lines. Series were created by dissecting 28-day-old rosettes and arranging the individual leaves. Four independent transgenic lines of *amiR* constructs and control plants were dissected; one representative line is shown. **(B)** Picture showing the chlorotic and dwarf phenotype and pigment content of *amiRT* compared to control lines. Chlorophyll *a* (Chl *a*), chlorophyll *b* (Chl *b*), and xanthophylls and carotenoids (X + C) were measured in four independent transgenic lines from 28-day-old plants. Each data point represents the mean value ± SD of eight biological replicates. For the leaf and cell area analyses, leaves from 21-day-old control and three independent lines of *amiRT* were incubated with lactic acid at room temperature until tissues were completely cleared and leaf epidermal and parenchymal cells were observed using differential interference contrast microscopy. Representative pictures and the measured areas of leaves **(C,F)**, epidermal **(D,G)**, and parenchymal **(E,H)** cells of the control and the triple *amiR* are shown. Data points represent mean areas of six leaves and a minimum of 650 epidermal and parenchymal cells per *amiR* or control lines. Asterisks indicate significance by two-sided *t*-test with *P < 0.01 and **P < 0.05.

Expanded rosettes of 28-day-old plants were dissected to better evaluate leaf phenotype in all knock-down lines (Figure 4A). Both single *amiR23.5* and *amiR23.6* and *amiR23.5/23.6* produced curved down leaves, unlike control plants with flat and completely expanded leaves. Leaves of *amiR26.5* did not display any difference to control leaves. On the contrary, the leaves of *amiRT* lines showed small size, indicating an alteration in the plants’ correct development and growth. Besides, the *amiR23.5/23.6/26.5* plants produced chlorotic and reticulated leaves (Figure 4B) in all independent lines analyzed. Therefore, we determined the photosynthetic pigments in the leaf of these plants (Figure 4B). Among the quantified pigments, the levels of chlorophyll *a* showed a significant reduction in all *amiRT* lines (Figure 4B). Chlorophyll *b* and xanthophylls plus carotenoid contents, however, exhibited statistically significantly lower levels only in one line each (*amiRT-3* and *amiRT-1*, respectively).

In light of the small plant size observed in the *amiRT* lines, leaf cells from these *amiRT* and control lines were observed using differential interference contrast microscopy, and their areas were measured. Besides the reduced leaf areas (Figures 4C,F), epidermal (Figures 4D,G) and parenchymal (Figures 4E,H) cell areas were significantly smaller in the triple knock-down line. However, the estimated number of epidermal and parenchymal cells per leaf showed no significant differences between the *amiRT* and control plants (data not shown), suggesting that the leaves’ phenotype might be due to the smaller cell size but not to a less number of them.

### *amiR* Lines Showed Reduced Photosynthetic Efficiency

**FIGURE 5:**
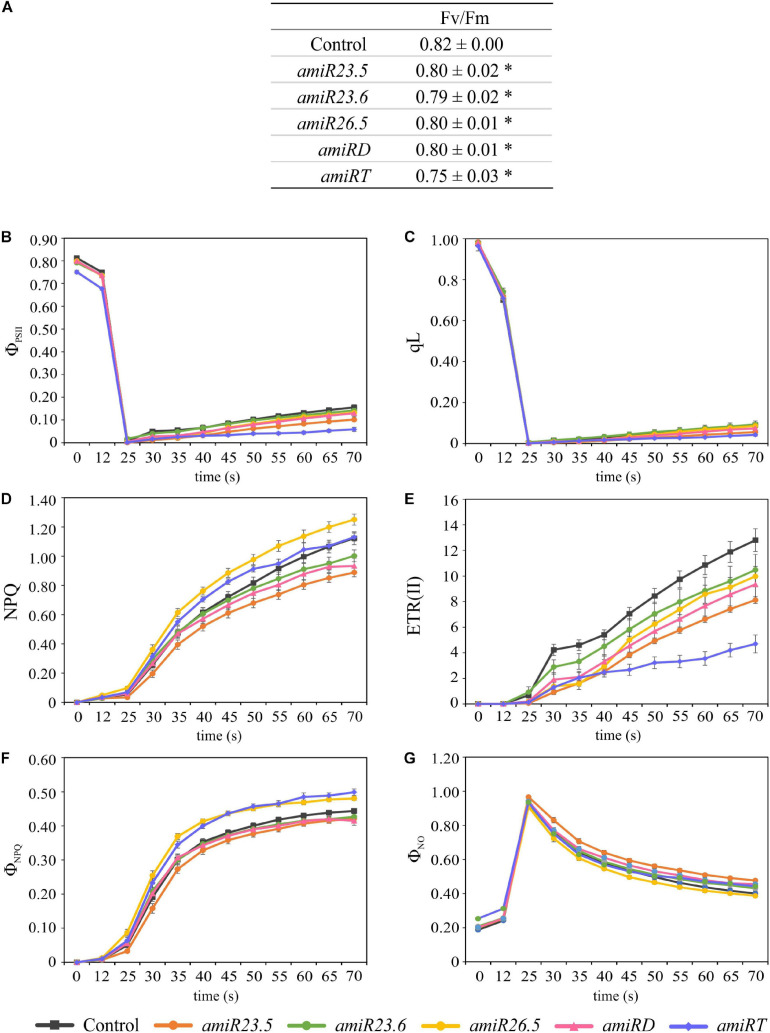
Photosynthetic response in control and *amiR* lines. Plants were grown for 21 days, dark adapted for 2 h and used for PAM fluorometric measurements. The Fv/Fm **(A)**, Φ_PSII_ **(B)**, qL **(C)**, NPQ **(D)**, ETR(II) **(E)**, Φ_NPQ_ **(F)**, and Φ_NO_ **(G)** parameters were evaluated during 70 s. An induction protocol with actinic light of 166 μmol photons m^–2^ s^–1^ and saturating pulses every 5 s was applied to individual leaves. The data show variations of the different photosynthetic parameters over time after the first 25 s up to 70 s. In each construct, four independent transgenic lines and a minimum of three replicates per line were measured. Twelve control plants were also evaluated. The data were combined in each *amiR* line and used in paired comparisons with the control data. The experiment was repeated twice, data shown correspond to one representative experiment. The data represent the mean value ± SE of a minimum of 12 biological replicates in every line. Asterisks in panel **(A)** indicate significance by two-sided *t*-test with *P < 0.01.

To monitor the photosynthetic performance of the plants, we used a PAM fluorometer. Plants (21-day-old control and *amiR* lines) were previously dark adapted, and then illuminated and saturating pulses of illumination were applied every 5 s to measure the photosynthetic parameters. After 25 s of illumination, *amiR* lines showed clear alteration of all photosynthetic parameters. Maximal efficiency of PSII (Fv/Fm) was significantly lower in all *amiR* lines compared to control plants (Figure 5A), showing that *amiRT* has the lowest value. Two parameters related to the PSII efficiency, the quantum yield of PSII (ΦPSII) and the proportion of photochemically active PSII reaction centers (qL), were also evaluated after illumination. After 40 s, ΦPSII and qL values decreased in *amiR* lines compared to control plants, and *amiRT* exhibited significantly lower ΦPSII and qL levels during the complete induction measurement (Figures 5B,C). These results indicate a less efficient PSII in *amiR* lines with the exception of *amiR23.6*, which showed control like ΦPSII and qL values. Related to this, the electron transport rate, ETR(II), significantly decreased in *amiR23.5*, *amiR26.5*, *amiRD*, and *amiRT* (Figure 5E). It is worth mentioning that lower PSII efficiency and ETR in *amiR* lines could lead to alterations in the CO_2_ assimilation of the *amiR* plants. Another estimated parameter was the non-photochemical quenching (NPQ), the apparent rate constant for heat loss from PSII. NPQ significantly decreased in *amiR23.5* and increased in *amiR26.5* compared to control plants (Figure 5D). NPQ was higher in *amiRT* at the beginning of illumination but then reached control levels (Figure 5D). Although no significant differences were found in *amiR23.6* and *amiRD*, NPQ exhibited a clear reduction in both lines, suggesting lower efficiency in the non-photochemical energy dissipation. In addition, other NPQ-related parameters, the light-induced NPQ (ΦNPQ), and the basal dissipative pathways (ΦNO) were estimated during light induction (Figures 5F,G). *amiR26.5* and *amiRT* showed significantly higher ΦNPQ, while in *amiR23.5*, ΦNPQ decreased and ΦNO increased compared to control values. In *amiR23.6* and *amiRD*, the values of these two parameters were similar to control plants (Figures 5F,G).

### *M-sHSPs* Impact on the Reproductive Growth

All *amiR* plants showed no evident differences in the aspect of flowers and siliques compared to control plants. Nevertheless, the seed area was significantly reduced in the single *amiR23.6* and in the *amiRD* and *amiRT* lines (Supplementary Figure 4A), indicating that the presence of M-sHSP23.6 is necessary for the seed to acquire the proper size. The seed yield was similar to control in all *amiR* lines, except for the *amiRT*, where it was significantly reduced (Supplementary Figure 4B). Seed germination was also affected in the *amiRT*; it was highly reduced (more than 90%) in comparison with control seeds (Supplementary Figure 4C). In the *amiRD* and single *amiR23.5* and *amiR23.6* lines, seed germination was significantly reduced (ca. 20%), while in *amiR26.5*, it was not affected.

### Almost All *amiR* Plants Accumulated ROS, and the Cell Membrane Permeability Increased

The level of ROS was examined in adult plants by standard histochemical detection. Accumulation of H_2_O_2_ was found in leaves of *amiR23.5* and *amiR23.6*, and in the double *amiR23.5/23.6* plants, which can be visualized as dark brown color by staining with DAB (Supplementary Figure 5A). On the other side, a reduced DAB staining was observed in the leaves of *amiR26.5* and *amiRT* plants, indicating less H_2_O_2_ than in the control leaves. The level of O_2_^.–^ in the leaves was detected by using NBT (Supplementary Figure 5B). Results show that *amiRT* lines seem to actively accumulate O_2_^⋅_–_^, leading to a deep blue coloration, while *amiR26.5* has reduced O_2_^.–^ levels (Supplementary Figure 5B). To visualize cell death in *amiR* lines, trypan blue staining was performed (Supplementary Figure 5C). While in all single and double knock-down lines, trypan blue staining did not show an accumulation, deep blue staining was detected in the *amiRT* leaves, considerably higher than in control leaves (Supplementary Figure 5C). These data suggest that the lower number of functioning cells in *amiRT* lines is related to the lower H_2_O_2_ level in *amiRT* leaves. Overall, these data indicate higher levels of O_2_^.–^ and a greater degree of cell death in the *amiRT* leaves, while *amiR26.5* leaves showed lower accumulation of ROS, H_2_O_2_, and O_2_^.–^.

High levels of ROS can be extremely harmful to cells by causing lipid peroxidation in cellular membranes, protein and carbohydrate oxidation, DNA damage, and cell death (Gill and Tuteja, 2010). The ion permeability of cell membranes, which is considered an indicator of cell damage induced by ROS, significantly increased in all *amiR* lines, showing higher electrolyte leakage than control plants (Supplementary Figure 6), indicating the loss of cell membrane stability and integrity. Nevertheless, the *amiR26.5* plants showed higher electrolyte leakage, which was not related to their leaves’ ROS levels (Supplementary Figure 5).

### Downregulation of *M-sHSP*s Produced Dramatic Changes in the Proteome

**FIGURE 6:**
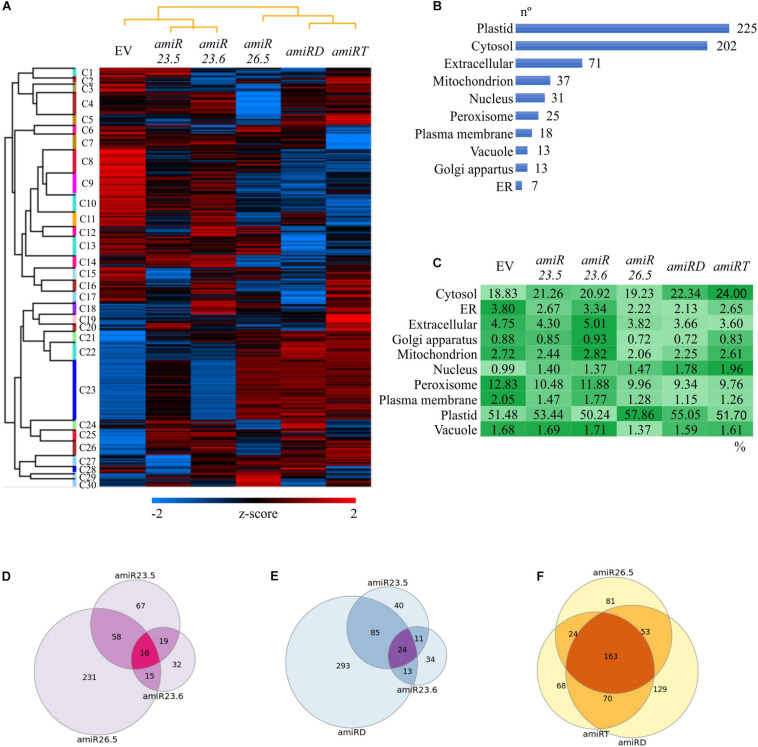
Proteome alterations in the *amiR* plants. **(A)** Hierarchical clustering of *amiR* and control (EV) proteomes. An average linkage clustering of proteins was performed using a Euclidian distance method for the *z* score normalized abundances (Supplementary Table 4). The data were filtered to have three valid values in at least one sample group. A raw tree for proteins and a column tree for samples were performed. Colors indicate intensities from the lowest (blue) to the highest (red). **(B,C)** Subcellular localization of differentially expressed proteins in the *amiR* lines. The list of statistically changed proteins in at least one *amiR* line (Supplementary Table 5) was analyzed by SUBA 4 (Hooper et al., 2017) and classified in 10 main subcellular categories; the number of proteins in each of them is shown **(B)**. **(C)** The protein abundance in each subcellular localization was calculated (Supplementary Table 11) and shown as a comparative color gradient, from the highest (darker green) to the lowest (lightest green). **(D–F)** Venn diagrams showing common and unique altered proteins in the knock-down lines. The differentially expressed proteins in different *amiR* lines (Supplementary Tables 6–10) were imported into the Venndiagram.net website, and the intersections between proteomes were graphed. The common and exclusive proteins found in the three single *amiR* **(D)**; *amiR23.5*, *amiR23.6*, and *amiRD* **(E)**; and *amiR26.5*, *amiRD*, and *amiRT* **(F)** are shown. All proteins are listed in Supplementary Tables 12–14.

Protein extractions from *amiR* and empty vector control plants were performed in 15-day-old seedlings, the single vegetative stage where no phenotypic differences were observed (Figure 3A). Comparable protein yields were obtained in control and *amiR* samples, as revealed by the protein quantitation and SDS polyacrylamide gel of the samples indicating that the *M-sHSPs* downregulation did not affect the total protein content. Samples were further analyzed by comparative quantitative shotgun MS/MS. A total of 2414 protein groups with at least two unique peptides were identified in the complete dataset (Supplementary Tables 2, 3). The processed LFQ data were filtered to have three valid values in at least one sample group, and the resulting 1290 protein matrix was used to perform a hierarchical clustering (Supplementary Table 4). In total, 30 clusters were defined and presented as a heat map in Figure 6A. A visible and profound alteration in the overall proteome is observed in all *amiR* lines. Specific groups of proteins accumulated in the single *amiR* plants, such as in Cluster 1 (in *amiR23.5*), Clusters 4 and 18 (in *amiR23.6*), and Clusters 6, 29, and 30 (in *amiR26.5*). Cluster 28 and Cluster 5 grouped proteins abundant in *amiRD* and in *amiRT*, respectively. Specific clusters with a lower abundance of proteins compared to control are observed in the *amiR26.5* (Clusters 3–5), *amiRD* (Clusters 12 and 17), and *amiRT* (Cluster 7).

To identify changes in specific proteins, *amiR* samples were individually compared to the control proteome, and the proteins with statistically significant alterations in at least one *amiR* line (642) were filtered (Supplementary Table 5). The individual proteins changed in each *amiR* line are listed in Supplementary Tables 6–10. The lower number of proteins identified in the *amiR23.6* proteome is related to detection issues. The 642 proteins were investigated for the subcellular localization by SUBA 4 (Hooper et al., 2017), classified in 10 categories (Figure 6B), and the relative protein abundance (percentage of the total LFQ of the 642 proteins) was calculated (Figure 6C and Supplementary Table 11). Interestingly, the highest number of changing proteins (225) are localized in the plastid, which are especially abundant in *amiR26.5* (57.86%), followed by the cytosolic proteins (202), more abundant in *amiRT* (24%). The *amiR23.6* and control plants exhibited the most similar protein abundances in each localization. The relative protein abundances of cytosol, nucleus, and plastid of the other *amiR* lines are higher than control lines, while compartments such as endoplasmic reticulum, extracellular, mitochondrion, and peroxisome showed lower abundances of proteins compared to the control proteome (Figure 6C). These ubiquitous changes indicate that mitochondria are not the only affected organelle when *M-sHSP*s are downregulated.

The lists of significantly changed proteins (Supplementary Tables 6–10) were further compared to found common and unique proteins in the knock-down lines. The single *amiR* lines have 16 proteins in common with members from almost all cell compartments (Figure 6D and Supplementary Table 12), but with a high number of unique proteins. The *amiR26.5* proteome was notably different from those in the other single *amiR* lines with 231 unique proteins. Since *amiR23.5* and *amiR23.6* exhibited similar phenotypic alterations, which are also observed in the *amiRD*, their proteomes were compared. These three lines shared 24 proteins (Figure 6E and Supplementary Table 13), whereas 293 unique proteins differentiate the *amiRD* proteome from the single *amiR*. These data suggest that new cell alterations may occur when both *sHSPs* are simultaneously downregulated in *amiRD*. Finally, the triple *amiRT*, the double *amiRD*, and the *amiR26.5* proteomes were analyzed (Figure 6F and Supplementary Table 14). The *amiRT* proteome showed most of its proteins in common with *amiRD* and *amiR26.5* proteomes, and only 68 unique proteins, despite the distinct phenotype of the *amiRT* plants.

**FIGURE 7:**
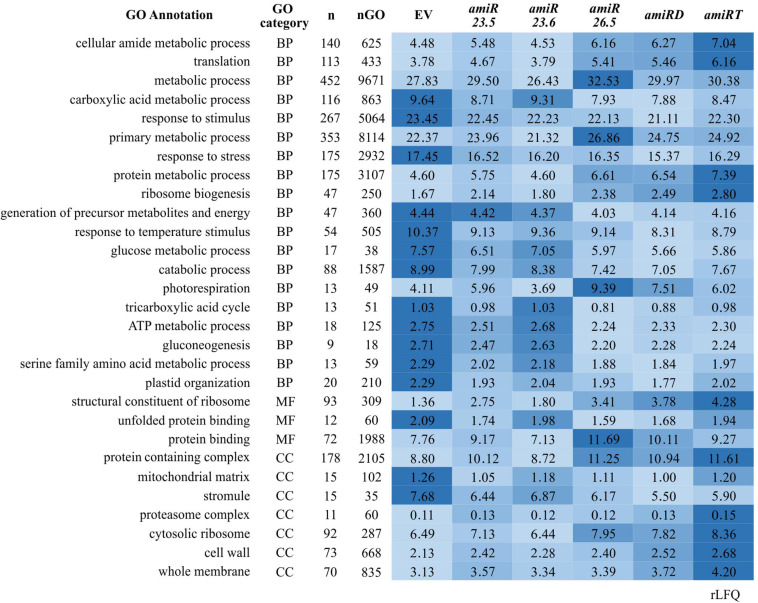
Comparative color gradient showing changes in the intensity of proteins involved in specific functions. Proteins that show statistical change relative to control (EV) plants in at least one *amiR* line (Supplementary Table 5) were analyzed by STRING (Szklarczyk et al., 2019) for the Gene Ontology (GO) classification. Selected GO terms in the biological process (BP), molecular function (MF), and cellular component (CC) categories are shown. n and nGO is the number of proteins found in the dataset and the total number of proteins in one GO, respectively. The relative abundance (rLFQ) of all proteins allocated to one GO was summed in each *amiR*. A color gradient from the highest rLFQ (darker blue) to the lowest (lightest blue) was used. Values correspond to the average of the total rLFQ for one GO in the three biological replicates.

To analyze the functional enrichments of the 642 differentially expressed proteins along the *amiR* lines (Supplementary Table 5), we applied a GO term analysis (Figure 7). The results revealed several enriched GO terms (Supplementary Table 15); the relative protein abundances (rLFQ) of selected GO terms were calculated and analyzed (Supplementary Table 16). Again, similar patterns in the protein abundances are found for the *amiR26.5*, *amiRD*, and *amiRT* (Figures 6A, 7). The protein abundance of *amiR23.6* showed the highest similarity to the control proteome. The abundances of proteins participating in cellular amide, protein binding, translation, protein degradation (proteasome complex), and metabolic processes are higher in the *amiR* than in control lines. Another point to highlight is the higher abundance of proteins involved in photorespiration found in the proteomes of *amiR* compared to control lines. In contrast, proteins related to sHSP functions such as response to stimulus and stress, including high temperature, and unfolding protein binding, are reduced in the *amiR* lines (Figure 7). The abundances of proteins from the mitochondrial matrix decreased in all *amiR*. Similarly, lower rLFQs were observed in stromule, plastid extensions probably involved in signaling and trafficking between plastids and other structures (Waters et al., 2004), whereas the abundance of proteins from the cell membrane and cell wall increased.

### *amiR* Plants Exhibited Alteration of Several Photosynthesis-Related Proteins

Due to the chlorotic phenotype of *amiRT* plants (Figure 4B) and the reduced photosynthetic efficiency of *amiR* plants (Figure 5A), the proteomes of these plants and single and double knock-down transgenic lines were analyzed for photosynthesis-related proteins (Table 1). The proteomes of *amiR23.5*, *amiR26.5*, *amiRD*, and *amiRT* showed enrichment of the GO photosynthesis (GO:0015979), but not the *amiR23.6* (Supplementary Tables 17–21). These data are in line with the photosynthetic parameter measurements, where *amiR23.6* line showed control like PSII efficiency (Figures 5B,C). Proteins involved in photosynthesis represent 7.49% and 9.31% of the total proteome in *amiRT* and *amiRD*, respectively (Supplementary Table 22). Proteins related to the stability of PSI and PSII, the efficiency of electron transfer, and oxygen evolution are highly accumulated in the *amiR26.5*, *amiRD*, and *amiRT* lines (Q9SA56, Q42029, Q9SHE8, Q9S714, and Q9XFT3) (Table 1). The Rubisco small subunit is also upregulated in the double and triple knock-down lines. The subunits A and B of the plastidial glyceraldehyde 3-phosphate dehydrogenase (Q9LPW0, P25857, and P25856) are significantly downregulated in the *amiR* lines compared to control plants as well as other two proteins involved in the carbon fixation (P25697 and O23404).

**TABLE 1:** Photosynthesis-related proteins in the *amiR* plants showing significant level change relative to control plants.

			*amiR 23.5*	*amiR 23.6*	*amiR 26.5*	*amiRD*	*amiRT*
			
Accession	Annotation	Protein code	log_2_ LFQ difference				
*AT1G03130.1*	Photosystem I subunit D-2 (PSAD-2)	Q9SA56	nsc	nsc	nsc	26.47	26.55
*AT1G06680.1*	Oxygen-evolving enhancer protein 2-1	Q42029	nsc	nsc	2.64	2.57	2.17
*AT1G12900.1*	Glyceraldehyde 3-phosphate dehydrogenase A subunit 2 (GAPA-2)	Q9LPW0	nsc	nsc	−0.55	−0.55	−0.56
*AT1G31330.1*	Photosystem I subunit F (PSAF)	Q9SHE8	nsc	nsc	nsc	25.63	25.60
*AT1G32060.1*	Phosphoribulokinase (PRK)	P25697	−0.29	nsc	nsc	−0.38	−0.40
*AT1G42970.1*	Glyceraldehyde-3-phosphate dehydrogenase B subunit (GAPB)	P25857	nsc	nsc	−0.23	−0.38	−0.40
*AT1G67090.1*	Ribulose bisphosphate carboxylase small chain 1A (RBCS1A)	P10795	nsc	nsc	nsc	5.29	4.25
*AT1G76450.1*	Photosystem II reaction center psbp family protein	Q9S720	nsc	nsc	nsc	nsc	24.09
*AT2G20260.1*	Photosystem I subunit E-2 (PSAE-2)	Q9S714	nsc	nsc	25.97	26.10	26.02
*AT3G26650.1*	Glyceraldehyde 3-phosphate dehydrogenase A subunit (GAPA)	P25856	−0.31	nsc	−0.36	−0.51	−0.55
*AT3G56090.1*	Ferritin 3 (FER3)	Q9LYN2	nsc	nsc	nsc	2.08	1.94
*AT3G56940.1*	Magnesium-protoporphyrin IX monomethyl ester [oxidative] cyclase	Q9M591	nsc	nsc	nsc	nsc	0.75
*AT3G63540.1*	Mog1/psbp/DUF1795-like photosystem II reaction center psbp family protein	P82658	nsc	nsc	nsc	24.91	24.67
*AT4G15530.5*	Pyruvate orthophosphate dikinase (PPDK)	O23404	−0.61	nsc	−1.13	−1.89	−1.18
*AT4G21280.2*	Oxygen-evolving enhancer protein 3-1	Q9XFT3	nsc	nsc	5.78	nsc	4.53
*AT5G13630.1*	Magnesium-chelatase subunit chlh,	Q9FNB0	0.24	nsc	nsc	nsc	0.67
*AT5G23120.1*	High chlorophyll fluorescence 136 (hcf136)	O82660	nsc	nsc	nsc	−0.42	−0.48
*AT5G38410.3*	Ribulose bisphosphate carboxylase (small chain) family protein	P10798	nsc	nsc	nsc	4.98	4.18
*AT5G38430.1*	Ribulose bisphosphate carboxylase (small chain) family protein	P10796	nsc	nsc	nsc	29.04	28.31
*AT1G12000.1*	Phosphofructokinase family protein	Q8W4M5	24.54	nsc	nsc	nsc	nsc
*AT1G61520.1*	Photosystem I light harvesting complex gene 3 (LHCA3)	Q9SY97	nsc	nsc	nsc	24.69	nsc
*AT2G47940.1*	Degp2 protease (DEGP2)	B3H581	−0.56	nsc	nsc	−0.53	nsc
*AT3G55330.1*	Psbp-like protein 1	P82538	nsc	nsc	25.58	nsc	nsc
*AT4G03280.1*	Photosynthetic electron transfer C	Q9ZR03	nsc	nsc	nsc	25.03	nsc
*AT4G27440.1*	Protochlorophyllide oxidoreductase B	P21218	1.07	nsc	0.854	1.37	nsc
*AT5G42270.1*	Variegated 1 (var1)	Q9FH02	−0.60	nsc	nsc	nsc	nsc

*Proteins differentially expressed in the knock-down plants relative to control plants (Supplementary Tables 17–21) were analyzed by using STRING (Szklarczyk et al., 2019) and filtered for the Gene Ontology term photosynthesis (GO:0015979). The values express the statistical differences >0.4 and <-0.4 in the log_2_-transformed LFQ intensities in each *amiR* line relative to control samples (two-sided *t-*test, *P* < 0.05). Differences with no statistical significance were excluded from the table and are shown as nsc.*

### The *amiR* Plants Showed Significant Changes in Their ROS-Related Proteins

Several proteins with essential roles in ROS detoxification, including chloroplast thiol-specific peroxidases that catalyze the reduction of H_2_O_2_, were upregulated in the *amiR26.5* plants (Table 2). The Fe-superoxide dismutase 1 (FSD1), which removes O_2_^⋅_–_^, also increases in *amiR26.5* lines but not in others. Single knock-down *amiR23.5* and *amiR23.6* did not exhibit significant increases in these proteins, while catalase and the chloroplastic glutathione reductase (P42770) decreased in the *amiR23.5* plants. Proteins related to oxidative stress were also upregulated in the proteome of *amiRD* plants such as the peroxiredoxins type 2, Q (PRXQ) and IIF, and the thioredoxin M type 4. In addition to the two downregulated proteins (catalase and glutathione reductase) in the *amiR23.5*, the *amiRD* showed a reduction of Fe-superoxide dismutase 2. The *amiRT* lines accumulate three chloroplastic peroxiredoxins (Q949U7, Q9LU86, and Q9C5R8) and a cytosolic glutathione transferase as in *amiR26.5*, whereas four secreted forms of peroxidases slightly decreased in these plants (Table 2). The statistically changed proteins in each *amiR* that are ROS-related (GO:0045454 and GO:0006979) represent, on average, 0.97%, 1.41%, 1.19%, and 1.34% of the total proteome of *amiR23.5*, *amiR26.5*, *amiRD*, and *amiRT*, respectively (Supplementary Table 23). None of these GO terms showed enrichment in the *amiR23.6* (Supplementary Table 18). This increased antioxidant capacity in the single *amiR26.5* and *amiRT* plants could alleviate, in part, the ROS accumulation (Supplementary Figure 5). Indeed, the histochemical ROS detection showed no accumulation of H_2_O_2_ and O_2_^.–^ molecules in the *amiR26.5* and H_2_O_2_ in the *amiRT* leaves.

**TABLE 2:** Proteins with antioxidant activity in the *amiR* plants showing significant level change relative to control plants.

				*amiR 23.5*	*amiR 23.6*	*amiR 26.5*	*amiRD*	*amiRT*
				
Accession	Annotation	Subcellular location	Protein code	log_2_ LFQ difference				
*AT3G52960.1*	Peroxiredoxin type 2	Plastid	Q949U7	nsc	nsc	28.35	28.29	28.10
*AT3G26060.2*	Peroxiredoxin Q (PRXQ)	Plastid	Q9LU86	nsc	nsc	26.75	26.90	25.91
*AT5G06290.1*	2-Cys peroxiredoxin (2-Cys prxb)	Plastid	Q9C5R8	nsc	nsc	26.19	nsc	25.67
*AT3G15360.1*	Thioredoxin M-type 4	Plastid	Q9SEU6	nsc	nsc	26.09	25.66	nsc
*AT3G06050.1*	Peroxiredoxin IIF	Mitochondrion	Q9M7T0	nsc	nsc	nsc	24.89	24.95
*AT4G03520.1*	Thioredoxin M2	Plastid	Q9SEU8	nsc	nsc	24.31	nsc	nsc
*AT3G11630.1*	2-Cys peroxiredoxin (2-Cys prxa)	Plastid	Q96291	nsc	nsc	5.31	nsc	nsc
*AT4G25100.1*	Fe-superoxide dismutase 1 (FSD1)	Plastid	P21276	nsc	nsc	2.39	nsc	nsc
*AT1G78380.1*	Glutathione s-transferase TAU 19	Cytosol	Q9ZRW8	nsc	nsc	2.30	nsc	2.20
*AT1G19570.1*	Dehydroascorbate reductase	Peroxisome	Q9FWR4	nsc	nsc	2.15	2.52	nsc
*AT5G17820.1*	Peroxidase 57 (PRXR10)	Extracellular	Q43729	nsc	nsc	nsc	1.48	nsc
*AT2G38380.1*	Peroxidase 22 (PRXEA)	Extracellular	P24102	0.93	0.77	nsc	0.80	0.66
*AT5G51100.1*	Superoxide dismutase [Fe] 2	Plastid	Q9LU64	nsc	nsc	nsc	−0.50	nsc
*AT5G64120.1*	Putative peroxidase	Extracellular	Q43387	nsc	nsc	nsc	nsc	−1.50
*AT5G64100.1*	Peroxidase 69	Extracellular	Q96511	nsc	nsc	nsc	nsc	−1.21
*AT4G35090.1*	Catalase (CAT2)	Peroxisome	P25819	−0.43	nsc	−0.33	−0.66	−0.63
*AT3G54660.1*	Glutathione reductase	Plastid	P42770	−0.40	nsc	nsc	−0.55	nsc
*AT3G21770.1*	Peroxidase 30 (PRXR9)	Extracellular	Q9LSY7	nsc	nsc	nsc	nsc	−23.68

*Proteins differentially expressed in the knock-down plants relative to control plants (Supplementary Tables 17–21) were analyzed by using STRING (Szklarczyk et al., 2019) and filtered for the Gene Ontology term antioxidant activity (GO:0016209). The statistical differences >0.4 and <-0.4 in the log_2_-transformed LFQ intensities in each *amiR* line relative to control samples are shown (two-sided *t*-test, *P* < 0.05). Differences with no statistical significance were excluded from the table and are shown as nsc.*

### Silencing of *M-sHSPs* Leads to Profound Metabolic Alterations

**FIGURE 8:**
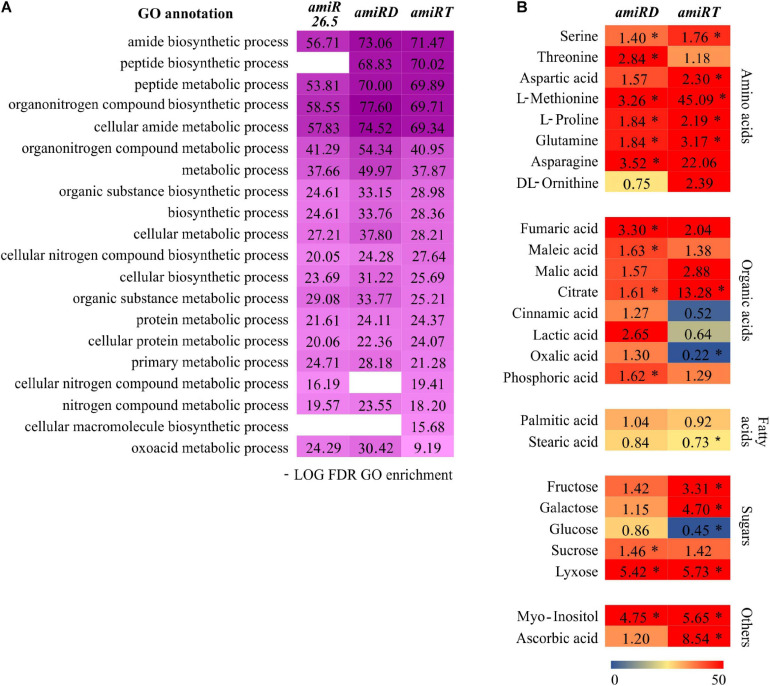
Metabolic changes in the *amiRD* and *amiRT* plants. **(A)** Color map showing top significant metabolism-related GOs in the differentially expressed proteins from *amiR26.5*, *amiRD*, and *amiRT* (Supplementary Tables 19–21). The -log_10_ of the calculated false discovery rate (FDR) is shown. **(B)** Metabolic profiling of *amiRD* and *amiRT* by GC-MS. The heat map shows the changes in the metabolome of *amiRD* and *amiRT* relative to control (EV) samples. Metabolites were extracted from 15-day-old plants and analyzed as described in Materials and Methods. Numbers indicate fold changes relative to control. Experiments were based on three biological replicates corresponding to three independent lines of each *amiR*; replicates consisted of a pool of 50 plants. Metabolites are represented by a color scale from blue (downregulated) to red (upregulated). Asterisks mean significance by a two-sided *t*-test with *P < 0.05.

The functional analysis of the changed proteins in the *amiR* plants (Supplementary Tables 6–10) showed the enrichment of various metabolic processes in the *amiR26.5*, *amiRD*, and *amiRT* (Supplementary Tables 19–21). These data were filtered to keep the first 20 most significant metabolism-GOs (Figure 8A). The highest overrepresentation was found in the double and triple knock-down lines in at least 16 GOs, indicating that a metabolic disruption may be more substantial in these lines (Figure 8A).

To further test the primary metabolism of *amiRD* and *amiRT* plants, metabolic profiling by GC-MS was performed. At this stage (15-day-old plants), *amiRD* and *amiRT* lines accumulated several amino acids, such as serine, methionine, glutamine, and proline (Figure 8B). Among the pool of organic acids, the double *amiR* showed significantly increased levels of fumaric, maleic, phosphoric, and citric acid, which showed a significant accumulation in the *amiRT*. Fructose and galactose highly accumulated in the *amiRT*, while lyxose significantly increased in *amiRD* and *amiRT* lines. It is worth mentioning the increment in the *amiRD* and *amiRT* lines of myo-inositol, a small molecule that is crucial in the regulation of growth and development (Donahue et al., 2010) in the *amiRT* plants.

### Downregulation of *M-sHSPs* Impacted the Mitochondrial Protein Profile in the *amiR* Lines

Several mitochondrial proteins (localization according to SUBA4 database) were differently affected in the *amiR* lines (Table 3). Also worth highlighting are the glycine cleavage system H protein 3 involved in glycine decarboxylation, associated with photorespiration in Arabidopsis (Eisenhut et al., 2019), and the ARM repeat superfamily protein, which was reported to interact with isocitrate dehydrogenase, an enzyme of the tricarboxylic acid cycle (Zhang et al., 2018), in *amiR26.5*, *amiRD*, and *amiRT* lines. The proteome of *amiRD* showed upregulation of several proteins related to the membrane respiratory chain NADH dehydrogenase (Complex I) and the 24-kDa subunit of the membrane ATP synthase; the peptidyl-prolyl cis-trans isomerase CYP19-3, which accelerates the folding of proteins; the haloacid dehalogenase-like hydrolase; and the peroxiredoxin-IIF, involved in the antioxidant activity (Table 2), also upregulated in *amiRT*.

**TABLE 3:** Mitochondrial proteins of *amiR* lines showing significant changes relative to the control plants.

			*amiR 23.5*	*amiR 23.6*	*amiR 26.5*	*amiRD*	*amiRT*
			
Accession	Protein code	Annotation	log_2_ LFQ difference				
*AT1G32470.1*	Q9LQL0	Glycine cleavage system H protein 3	ns	ns	25.70	26.18	25.96
*AT2G21870.1*	F4III4	Probable ATP synthase 24 kDa subunit	ns	ns	ns	24.33	ns
*AT2G33255.1*	Q8RYE9	Haloacid dehalogenase-like hydrolase (HAD) superfamily protein	ns	ns	ns	23.84 ↓	ns
*AT3G06050.1*	Q9M7T0	Peroxiredoxin-IIF	ns	ns	ns	24.88	24.95
*AT3G56070.1*	Q38867	Peptidyl-prolyl cis-trans isomerase CYP19-3	ns	ns	ns	24.20 ↓	ns
*AT3G59760.1*	Q43725	Cysteine synthase	nsc	16.25 ↓	ns	16.32 ↓	ns
*AT3G62530.1*	Q94K48	ARM repeat superfamily protein	ns	ns	25.32 ↓	25.50 ↓	25.48 ↓
*AT4G02580.1*	O22769	NADH-ubiquinone oxidoreductase 24 kDa subunit	ns	ns	ns	24.23 ↓	ns
*AT4G11010.1*	O49203	Nucleoside diphosphate kinase III	ns	ns	25.99	26.09	25.98
*AT4G40030.2*	P59169	Histone superfamily protein	ns	ns	26.13	26.07	25.74
*AT5G10860.1*	Q9LEV3	CBS domain-containing protein CBSX3	ns	ns	25.08	24.53	ns
*AT5G26780.3*	Q94C74	Serine hydroxymethyltransferase 2	16.17 ↓	ns	ns	ns	ns
*ATMG00070.1*	Q95748	NADH dehydrogenase subunit 9	ns	ns	ns	24.50	ns

*Proteins differentially expressed in the *amiR* lines compared to control samples (two-sided *t*-test, *P* < 0.05) were analyzed by SUBA 4 (Hooper et al., 2017) for the subcellular localization and the mitochondrial proteins were identified. The values express the statistical differences in the log_2_-transformed LFQ intensities in each *amiR* line relative to the control samples in normal conditions (Supplementary Tables 5–10). Differences with no statistical significance are indicated as ns. The abundance of the same proteins was also analyzed in the *amiR* lines after heat stress (3 h at 37°C, Supplementary Tables 24–30). Arrows indicate a decrease in the protein levels during heat stress (two-sided *t*-test between normal and heat stress condition in each *amiR* line, *P* < 0.05). Changes with no statistical significance were excluded from the table.*

To test the effect of heat stress (3 h at 37°C) on the mitochondrial protein profile, the *amiR* lines’ proteome was also evaluated in 15-day-old plants. Most of the detected mitochondrial proteins decrease their level under heat stress (Table 3), while many cytosolic proteins related to heat response were upregulated; they are described below.

### Heat Conditions Mainly Affected the Cytosolic Protein Profiles in the *amiR* Lines

The proteome of the *amiR* lines was evaluated in 15-day-old plants under heat conditions (3 h at 37°C). Comparisons were established in empty vector control or *amiR* line separately, between heat and normal conditions (Supplementary Tables 24–30). After heat stress, the proteome of all lines showed enrichment of GO terms related to stress response, and *amiRD* exhibited the highest overrepresentation among *amiR* lines (Table 4). Proteins of different subcellular compartments (localization according to SUBA4 database) were differently affected by the heat condition in empty vector control and each *amiR* line (Supplementary Table 31). Although mitochondrial proteins were reduced in all lines, they were particularly affected in the *amiR23.5*, *amiR23.6*, *amiRD*, and *amiRT*. Cytosolic proteins highly accumulated after heat stress, reaching the highest level in *amiR23.6*, *amiRD*, and *amiRT*, while proteins from the rest of the compartments decreased their abundances in all samples (Supplementary Table 31). These results indicate an utterly new adjustment in the proteome homeostasis under high temperature.

**TABLE 4:** HSP superfamily proteins and stress-related GOs showing significant changes after heat stress in control and *amiR* lines.

		*Control*	*amiR 23.5*	*amiR 23.6*	*amiR 26.5*	*amiRD*	*amiRT*
		
Accession	Annotation	log_2_ LFQ difference					
*AT1G07400.1*	HSP17.8	27.49	28.46	28.02*	28.19	29.00	28.05
*AT1G16030.1*	HSP70-B	2.21	2.70	2.63	2.26	2.57	1.83
*AT1G53540.1*	HSP17.6C	26.13	28.40	–	27.52	28.74	27.03
*AT1G54050.1*	HSP17.4B	–	27.97	–	27.63	28.19	27.09
*AT1G59860.1*	HSP17.6A	–	26.25	–	25.67	26.22	25.71
*AT2G29500.1*	HSP17.6B	–	27.53	–	27.16	27.91	26.68
*AT2G32120.1*	HSP70-8	27.69	27.99	–	27.81	28.19	27.77
*AT3G09440.1*	HSP70-3	0.69	1.14	1.21	0.85	1.21	0.65
*AT3G12580.1*	HSP70-4	4.06	12.82	12.86	12.08	21.52	12.25
*AT3G46230.1*	HSP17.4A	26.43	27.93*	27.91	27.20	28.41	27.05
*AT4G10250.1*	HSP22.0 (ER)	26.04	27.03	–	26.67	27.59	26.71
*AT4G25200.1*	HSP23.6 (M)	27.24	28.49	–	28.15	–	–
*AT5G12030.1*	HSP17.7	24.54	–	–	25.16	25.00	–
*AT5G52640.1*	HSP90-1	30.57	22.89	22.89	30.35	30.99	29.89
*AT5G56030.2*	HSP81-2	0.64	0.94	1.06	0.96	1.09	0.68*
*AT2G26150.1*	HSFA2	–	24.97	–	24.42	25.08	26.35

		***Control***	***amiR 23.5***	***amiR 23.6***	***amiR 26.5***	***amiRD***	***amiRT***
		
**GO term**	**Description**	**−log_10_ FDR GO enrichment**					

GO:0009266	Response to temperature	19.42	18.74	9.87	18.90	20.12	12.29
GO:0006950	Response to stress	16.19	17.47	11.44	18.54	19.91	8.75
GO:0009408	Response to heat	7.30	12.56	8.38	12.18	14.01	9.79

*The abundance of members of the HSP superfamily was evaluated in control and *amiR* lines after heat stress (3 h at 37°C) and compared to their levels in control conditions (two-sided *t*-test between normal and heat stress condition in each line, *P* < 0.05, ^∗^<0.1). The values express the statistical differences in the log_2_-transformed LFQ intensities of the proteins after the heat stress treatment. ER, endoplasmic reticulum, M, mitochondria. The second table shows the enrichment of stress-related GOs in the proteome of control and *amiR* lines after heat stress. FDR, false discovery rate.*

From the three M-sHSPs, M-sHSP23.6 is the only one that was well detected with the proteomic approach, while M-sHSP23.5 and M-sHSP26.5 showed no consistent intensities in the analysis (Supplementary Table 32). M-sHSP23.6 exhibited significant induction under heat treatment in the proteome of all samples of control, *amiR23.5*, and *amiR26.5* plants, and in one or two samples of *amiRD* and *amiRT*, respectively. Under these conditions, all plants (control and all *amiR* lines) induced several cytosolic and one endoplasmic reticulum-localized HSPs in common (Table 4). However, a member of Heat Stress Transcription Factor (HsfA2, *At2g26150*) and members of cytosolic HSP20-like chaperone superfamily proteins (*At1g54050, At1g59860.1*, and *At2g29500*) were induced by heat in *amiR23.5*, *amiR26.*5, *amiRD*, and *amiRT*, but not in control plants (Table 4 and Supplementary Tables 25–30). Besides, the most substantial effect in the abundance of the detected HSP family members was observed in the *amiR23.5*, *amiR26.5*, and *amiRD*. These data indicate that the absence of any *M-sHSPs* triggered a distinct pattern of cytosolic response to heat stress.

## Discussion

Our understanding of how plant sHSPs function comes from studies that use overexpression strategies. Most of these studies were focused to enhance the heat tolerance of crops. Recently, plants overexpressing the mitochondrion-targeted Hsp24.7 (an ortholog of Arabidopsis *M-Hsp23.6*) of *G. hirsutum* were shown to enhance germination in transgenic cotton, Arabidopsis, and tomato (Ma et al., 2019). The aim of this work was to uncover the function of M-sHSPs in Arabidopsis plants growing under normal conditions. We present evidence that the simultaneous downregulation of all three Arabidopsis M-sHSPs is essential for plant growth and development under normal growing conditions. This is based on the phenotypic and molecular analyses of the *M-sHSPs* knock-down plants. Downregulation of the three *M-sHSPs* leads to a drastic disruption in the vegetative and reproductive growth. Triple *amiR23.5/23.6/26.5* plants are dwarf (Figures 3B–E) probably due to the small cell size (Figures 4C–H), with shorter roots (Figures 3F,G), have small pale and chlorotic leaves (Figures 4A,B), and produce a smaller and lower amount of seeds, with reduced germination rates (Supplementary Figure 4). The phenotype of the single and double knock-down lines contrast with the more prominent phenotype of the *amiR23.5/23.6/26.5* plants (Figure 3). The single *amiR23.5*, *amiR23.6*, and the double *amiRD* developed synergistic phenotypes, with curved down leaves, bigger rosettes, and plants (Figures 3, 4), and lower seed area and germination rate (Supplementary Figure 4C). Previous reports showed that *sHSP23.5* and *sHSP23.6* appear to be dual targeted to mitochondria and chloroplasts and were highly co-expressed (Van Aken et al., 2009). The authors suggested that sHSP23.5 and sHSP23.6 form a functional pair and both are needed for stabilizing mitochondrial proteins, and possibly plastidic proteins during stress conditions. As said before, M-sHSP23.5 and M-sHSP23.6 protein sequences are highly similar (68%), while they share only 33% similarity with M-sHSP26.5 (Supplementary Figure 1B). It is important to remark that the seed germination of the *amiR23.5* and *amiR23.6* lines opposed to the phenotype of *AtHSP23.6* overexpression lines that exhibited fast germination rates (Ma et al., 2019). Moreover, the seed phenotype of *amiR26.5* plants is indistinguishable from the control lines, indicating that M-sHSP26.5 is not essential for seed development and germination, but the absence of all three M-sHSPs makes a drastic effect on seed viability. The transcriptional upregulation of the *M-sHSPs* in the single and double knock-down lines lacking one or two of them (Figure 2) denotes that these proteins are strictly regulated. These data suggest that any of the others could compensate for the absence of one M-sHSP, but the absence of the three M-sHSPs causes severe plant growth and reproduction impairment.

Under normal conditions, the promotor of *M-sHSP26.5* was mainly active in the root of 15-day-old plants (Figure 1). At the same growth stage, the *amiR26.5* and *amiRT* plants exhibited a disruption in the root growth (Figure 3). It was previously suggested that the cell size is relevant in determining root length (Aceves-García et al., 2016). Nevertheless, the single *amiR26.5* showed reduced root length, without consequences in the plant growth or reproduction. Another thing to highlight is the lower protein abundance in *amiR26.5* and *amiRT* compared to control plants, of three subunits of the plastid-located glyceraldehyde 3-phosphate dehydrogenase (Table 1), and the cytosolic actin 2 (Q96292, *At3g18780.2*) (Supplementary Tables 5, 8, 10), with both proteins related to root growth (Muñoz-Bertomeu et al., 2009; Vaškebová et al., 2018). In fact, the phenotype of all double mutants of *gapc* showed a severe arrest of root development after 10 to 12 days of growth (Muñoz-Bertomeu et al., 2009), similar to the phenotype of *amiR26.5* and *amiRT* after 15 days of growth (Figures 3F,G). Additionally, *amiRT* showed downregulation of two putative peroxidases (Q43387 and Q96511) (Table 2) in coincidence with the downregulation of the expression of the genes (*At5g64120* and *At5g64100*) in the double mutants of *gapc*. It was postulated that glyceraldehyde 3-phosphate dehydrogenase could act as a plastid redox sensor (Muñoz-Bertomeu et al., 2009).

Concerning ROS homeostasis, several proteins from intracellular and extracellular compartments that participate in the antioxidant response accumulated in the single *amiR26.5*, such as peroxiredoxin and thioredoxin superfamily proteins, Fe superoxide dismutases, and peroxidases (Table 2). The important role of these proteins in the redox regulation of chloroplast target proteins was known. In particular, 2-Cys peroxiredoxins were found to be associated with the [Fe] superoxide dismutase, which are essential for the maintenance of plastidial redox homeostasis, due to the dismutation of superoxide radicals produced at the level of photosystem I to hydrogen peroxide (Cerveau et al., 2016). The proportion of the total proteome involved in the oxidative stress response and redox homeostasis increased in the *amiR26.5* when considering the protein abundances (Supplementary Table 23). The overrepresentation of proteins related to the antioxidant activity (GO:0016209) (Table 2) suggests that both mitochondrial and chloroplast ROS are detoxified, resulting in the lower level of ROS in the *amiR26.5* plants (Supplementary Figure 5).

The photosynthetic performance of *amiR* was affected in all lines compared to control plants, although in different ways (Figure 5). *amiRT* lines, which presented a high chlorotic phenotype (Figure 4), showed the lowest PSII efficiency and ETR values (Figures 5B,E). Proteins related to photosynthesis were significantly overproduced in *amiR26.5*, *amiRD*, and *amiRT* (Table 1). Specifically, the photosystem II reaction center psbp family protein was overproduced exclusively in the *amiRT*, while other PSI and photosynthetic electron transport proteins were overproduced in *amiR26.5* and *amiRD*. The alterations in the PSII efficiency and ETR values of *amiR* plants indicate that CO_2_ assimilation of the *amiR* plants is also affected. In fact, Rubisco small subunit family proteins were similarly upregulated in *amiRD* and *amiRT* plants (Table 1 and Supplementary Tables 9, 10). It is worth mentioning that a cpHsp70 (Q9STW6), involved in protein import into chloroplasts during early developmental stages (Su and Li, 2010), was significantly downregulated in *amiRD* and *amiRT* (Supplementary Tables 5, 9, 10), suggesting that the accumulation of the photosynthetic proteins could be due to an impairment of chloroplast protein import in these plants.

The *amiRT* significantly increased the production of primary metabolites in 15-day-old-plants (Figure 8B), indicating a metabolic reprogramming in these plants. Proline accumulation was reported to be detrimental for plant growth (Lv et al., 2011), which could contribute, in part, to the *amiRT* phenotype. Myo-inositol and galactose, precursors for the ascorbic acid biosynthesis (Lorence et al., 2004), and ascorbic acid (Figure 8B) are increased in 15-day-old *amiRD* and *amiRT* plants in comparison with the control lines, indicating a need to regulate the ROS production in mitochondria. Related to this, the malate content is highly increased in *amiRT* compared to control plants (Figure 8B). Malate could link the chloroplast metabolism with the mitochondrial ROS, as it was recently proposed (Zhao et al., 2020). Malate plays a key role as an effective readout of the chloroplast redox status leading to the mitochondrial ROS production (Zhao et al., 2020).

Another aspect to consider is the role of M-sHSPs in the stabilization of proteins and membranes (Wang et al., 2004). Several proteins at the cell membrane and cell wall were increased in the *amiRT*, indicating an alteration of these structures (Figure 7). The disruption of cell membranes in the knock-down plants, indicated by the higher electrolyte leakage (Supplementary Figure 6), supports the view that M-sHSPs may also function as membrane protectants as it was postulated in another system by regulating membrane fluidity and preserving membrane structure and integrity (Tsvetkova et al., 2002).

Except for vacuole and Golgi apparatus, proteins from all cell compartments changed their abundance (Figure 6C), indicating an alteration in the overall protein homeostasis of the *amiR* plants. Besides the photosynthetic and antioxidant proteins and the associated metabolites, results revealed a complete reprogramming of several crucial pathways and biological processes in response to the absence of M-sHSPs (Figures 7, 8). These new adjustments demonstrate that, although located and playing essential roles in mitochondria, M-sHSPs may have functional interconnections with other cellular processes and structures outside these organelles.

From the proteome of the *amiR* lines under heat conditions, the M-sHSP23.6 is the only one that was well detected in the single *amiR23.5* and *amiR26.5*, although not in all samples of *amiRD* and *amiRT* (Supplementary Table 32). These data could be due to the detection limits of the LC/MS protein analysis. Some mitochondrial proteins were similarly upregulated in *amiR26.5*, *amiRD*, and *amiRT* (Table 3). Several of these mitochondrial proteins are involved in the mitochondrial electron transport, ATP synthesis, and photorespiration. None of them was affected by heat treatment. However, several proteins belonging to the cytosolic HSP chaperone superfamily and HSF2 (Table 4 and Supplementary Table 24) were markedly increased by the absence of any *M-sHSPs*, triggering a distinct pattern of cytosolic response to heat stress.

In conclusion, the simultaneous downregulation of the three *M-sHSPs* in Arabidopsis is critical for the proper development and growth of the plant. In response to M-sHSP deficiency, the plant regulates the photosynthetic efficiency, and the level of proteins involved in photosynthesis, the antioxidant system, and mitochondrial metabolism. The protein profile of subcellular compartments was altered, mainly the plastid, cytosol, and mitochondrion, affecting the whole plant cell. Most likely, M-sHSPs coordinate the function between these intracellular spaces to maintain the cellular homeostasis.

While the individual absence of one of the M-sHSP is not critical for the plant to grow and reproduce, possibly by partial compensation of the function by the other proteins, the *amiRT* is severely affected and unable to complete a healthy life cycle. The absence of M-sHSPs seems to affect the coordination of the cellular components for growth. This is the first demonstration that the three M-sHSPs, involved in stress responses, are essential for the normal vegetative and reproductive growth of Arabidopsis.

## Data Availability Statement

The mass spectrometry proteomics data have been deposited to the ProteomeXchange Consortium via the PRIDE (Perez-Riverol et al., 2019) partner repository with the dataset identifier PXD019603.

## Author Contributions

IF and EV conceived and designed the research and supervised the research work. ME designed and performed the experiments. EV wrote the research article. All authors interpreted the results, read, edited, and approved the manuscript.

## Conflict of Interest

The authors declare that the research was conducted in the absence of any commercial or financial relationships that could be construed as a potential conflict of interest.

## Footnotes

1 http://wmd3.weigelworld.org

## References

[B1] Aceves-García P. Álvarez-Buylla E. R. Garay-Arroyo A. García-Ponce B. Muñoz R. Sánchez M. P. (2016). Root architecture diversity and meristem dynamics in different populations of *Arabidopsis thaliana*. *Front. Plant Sci.* 7:858. 10.3389/fpls.2016.00858 27379140 PMC4910468

[B2] Cerveau D. Kraut A. Stotz H. U. Mueller M. J. Couté Y. Rey P. (2016). Characterization of the *Arabidopsis thaliana* 2-Cys peroxiredoxin interactome. *Plant Sci.* 252 30–41. 10.1016/j.plantsci.2016.07.003 27717466

[B3] Clough S. J. Bent A. (1998). Floral Dip: a simplified method for Agrobacterium-mediated transformation of *Arabidopsis thaliana*. *Plant J.* 16 735–743. 10.1046/j.1365-313x.1998.00343.x 10069079

[B4] Cox J. Mann M. (2008). MaxQuant enables high peptide identification rates, individualized p.p.b.-range mass accuracies and proteome-wide protein quantification. *Nat. Biotechnol.* 26 1367–1372. 10.1038/nbt.1511 19029910

[B5] Dabbaghizadeh A. Morrow G. Amer Y. O. Chatelain E. H. Pichaud N. Tanguay R. M. (2018). Identification of proteins interacting with the mitochondrial small heat shock protein Hsp22 of *Drosophila melanogaster*: implication in mitochondrial homeostasis. *PLoS One* 13:e0193771. 10.1371/journal.pone.0193771 29509794 PMC5839585

[B6] de Miguel N. Echeverria P. Angel S. (2005). Differential subcellular localization of members of the *Toxoplasma gondii* small heat shock protein family. *Eukaryot. Cell* 4 1990–1997. 10.1128/EC.4.12.1990-1997.2005 16339717 PMC1317493

[B7] Donahue J. Alford S. Torabinejad J. Kerwin R. E. Nourbakhsh A. Rayet W. K. (2010). The *Arabidopsis thaliana* Myo-inositol 1-phosphate synthase1 gene is required for Myo-inositol synthesis and suppression of cell death. *Plant Cell* 22 888–903. 10.1105/tpc.109.071779 20215587 PMC2861443

[B8] Eisenhut M. Roell M. −S. Weber A. P. M. (2019). Mechanistic understanding of photorespiration paves the way to a new green revolution. *New Phytol.* 223 1762–1769. 10.1111/nph.15872 31032928

[B9] Fernández-Bautista N. Domínguez-Núñez J. A. Moreno M. C. Berrocal-Lobo M. (2016). Plant tissue trypan blue staining during phytopathogen infection. *Bio Protoc.* 6:2078 10.21769/BioProtoc.2078

[B10] Gill S. Tuteja N. (2010). Reactive oxygen species and antioxidant machinery in abiotic stress tolerance in crop plants. *Plant Physiol. Biochem.* 48 909–930. 10.1016/j.plaphy.2010.08.016 20870416

[B11] Grimplet J. Wheatley M. D. Jouira H. B. Deluc L. G. Cramer G. R. Cushman J. C. (2009). Proteomic and selected metabolite analysis of grape berry tissues under well-watered and water-deficit stress conditions. *Proteomics* 9 2503–2528. 10.1002/pmic.200800158 19343710 PMC4090949

[B12] Halliwell B. Gutteridge J. M. C. (2015). *Free Radicals in Biology and Medicine*, 5th Edn New York, NY: Oxford University Press.

[B13] Hieno A. Naznin H. A. Inaba-Hasegawa K. Yokogawa T. Hayami N. Nomoto M. (2019). Transcriptome analysis and identification of a transcriptional regulatory network in the response to H_2_O_2_. *Plant Physiol.* 180 1629–1646. 10.1104/pp.18.01426 31064811 PMC6752916

[B14] Hooper C. M. Castleden I. Tanz S. K. Aryamanesh N. Millar A. H. (2017). SUBA4: the interactive data analysis centre for *Arabidopsis* subcellular protein locations. *Nucleic Acids Res.* 45 D1064–D1074. 10.1093/nar/gkw1041 27899614 PMC5210537

[B15] Huther C. M. Ramm A. Rombaldi C. V. Bacarin M. A. (2013). Physiological response to heat stress of tomato ‘Micro-Tom’ plants expressing high and low levels of mitochondrial sHSP23.6 protein. *Plant Growth Regul.* 70 175–185. 10.1007/s10725-013-9790-y

[B16] Jacob P. Hirt H. Bendahmane A. (2017). The heat-shock protein/chaperone network and multiple stress resistance. *Plant Biotechnol. J.* 15 405–414. 10.1111/pbi.12659 27860233 PMC5362687

[B17] Jiang C. Xu J. Zhang H. Zhang X. Shi J. Li M. (2009). A cytosolic class I small heat shock protein, RcHSP17.8, of *Rosa chinensis* confers resistance to a variety of stresses to *Escherichia coli*, yeast and *Arabidopsis thaliana*. *Plant Cell Environ.* 32 1046–1059. 10.1111/j.1365-3040.2009.01987.x 19422616

[B18] Klughammer C. Schreiber U. (2008). Complementary PS II quantum yields calculated from simple fluorescence parameters measured by PAM fluorometry and the Saturation Pulse method. *PAM Appl. Notes* 1 27–35.

[B19] Leister D. Wang X. Haberer G. Mayer K. F. Kleine T. (2011). Intracompartmental and intercompartmental transcriptional networks coordinate the expression of genes for organellar functions. *Plant Physiol.* 157 386–404. 10.1104/pp.111.177691 21775496 PMC3165886

[B20] Lisec J. Schauer N. Kopka J. Willmitzer L. Fernie A. R. (2006). Gas chromatography mass spectrometry-based metabolite profiling in plants. *Nat. Protoc.* 1 387–396. 10.1038/nprot.2006.59 17406261

[B21] Lorence A. Chevone B. I. Mendes P. Craig L. Nessler C. L. (2004). Myo-Inositol oxygenase offers a possible entry point into plant ascorbate biosynthesis. *Plant Physiol.* 134 1200–1205. 10.1104/pp.103.033936 14976233 PMC389944

[B22] Lv W. T. Lin B. Zhang M. Hua X. J. (2011). Proline accumulation is inhibitory to *Arabidopsis* seedlings during heat stress. *Plant Physiol.* 156 1921–1933. 10.1104/pp.111.175810 21670222 PMC3149957

[B23] Ma W. Guan X. Li J. Pan R. Wang L. Liu F. (2019). Mitochondrial small heat shock protein mediates seed germination via thermal sensing. *Proc. Natl. Acad. Sci. U.S.A.* 116 4716–4721. 10.1073/pnas.1815790116 30765516 PMC6410843

[B24] Morrow G. Tanguay R. M. (2015). *Drosophila melanogaster* Hsp22: a mitochondrial small heat shock protein influencing the aging process. *Front. Genet.* 6:103. 10.3389/fgene.2015.00103 25852752 PMC4360758

[B25] Muñoz-Bertomeu J. Cascales-Miñana B. Mulet J. M. Baroja-Fernández E. Pozueta-Romero J. Kuhn J. M. (2009). Plastidial glyceraldehyde-3-phosphate dehydrogenase deficiency leads to altered root development and affects the sugar and amino acid balance in *Arabidopsis*. *Plant Physiol.* 151 541–558. 10.1104/pp.109.143701 19675149 PMC2754643

[B26] Neuhoff V. Arold N. Taube D. Ehrhardt W. (1988). Improved staining of proteins in polyacrylamide gels including isoelectric focusing gels with clear background at nanogram sensitivity using Coomassie Brilliant Blue G-250 and R-250. *Electrophoresis* 9 255–262. 10.1002/elps.1150090603 2466658

[B27] Perez-Riverol Y. Csordas A. Bai J. Bernal-Llinares M. Hewapathirana S. Kundu D. J. (2019). The PRIDE database and related tools and resources in 2019: improving support for quantification data. *Nucleic Acids Res.* 47 D442–D450. 10.1093/nar/gky1106 30395289 PMC6323896

[B28] Rhoads D. M. White S. J. Zou Y. Muralidharan M. Elthon T. E. (2005). Altered gene expression in plants with constitutive expression of a mitochondrial small heat shock protein suggests the involvement of retrograde regulation in the heat stress response. *Physiol. Plant.* 123 435–444. 10.1111/j.1399-3054.2005.00473.x

[B29] Sanmiya K. Suzuki K. Egawa Y. Shono M. (2004). Mitochondrial small heat-shock protein enhances thermotolerance in tobacco plants. *FEBS Lett.* 557 265–268. 10.1016/S0014-5793(03)01494-7 14741379

[B30] Scarpeci T. E. Frea V. Zanor M. I. Valle E. M. (2017). Overexpression of AtERF019 delays plant growth and senescence, and improves drought tolerance in *Arabidopsis*. *J. Exp. Bot.* 68 673–685. 10.1093/jxb/erw429 28204526

[B31] Scarpeci T. E. Zanor M. I. Valle E. M. (2008b). Investigating the role of plant heat shock proteins during oxidative stress. *Plant Signal. Behav.* 3 856–857. 10.1007/s11103-007-9274-4 19704521 PMC2634396

[B32] Scarpeci T. E. Zanor M. I. Carrillo N. Mueller-Roeber B. Valle E. M. (2008a). Generation of superoxide anion in chloroplasts of *Arabidopsis thaliana* during active photosynthesis: a focus on rapidly induced genes. *Plant Mol. Biol.* 66 361–378. 18158584 10.1007/s11103-007-9274-4 PMC2758387

[B33] Scharf K. D. Siddique M. Vierling E. (2001). The expanding family of *Arabidopsis thaliana* small heat stress proteins and a new family of proteins containing α-crystallin domains (Acd proteins). *Cell Stress Chaperones* 6 225–237. 10.1379/1466-12682001006<0225:tefoat<2.0.co;2 11599564 PMC434404

[B34] Schauer N. Steinhauser D. Strelkov S. Schomburg D. Allison G. Moritz T. (2005). GCMS libraries for the rapid identification of metabolites in complex biological samples. *FEBS Lett.* 579 1332–1337. 10.1016/j.febslet.2005.01.029 15733837

[B35] Schwab R. Ossowski S. Riester M. Warthmann N. Weigel D. (2006). Highly specific gene silencing by artificial microRNAs in *Arabidopsis*. *Plant Cell* 18 1121–1133. 10.1105/tpc.105.039834 16531494 PMC1456875

[B36] Shevchenko A. Tomas H. Havliš J. Olsen J. V. Mann M. (2007). In-gel digestion for mass spectrometric characterization of proteins and proteomes. *Nat. Protoc.* 1 2856–2860. 10.1038/nprot.2006.468 17406544

[B37] Siddique M. Gernhard S. von Koskull-Döring P. Vierling E. Scharf K. D. (2008). The plant sHSP superfamily: five new members in *Arabidopsis thaliana* with unexpected properties. *Cell Stress Chaperones* 13 183–197. 10.1007/s12192-008-0032-6 18369739 PMC2673886

[B38] Song L. Jiang Y. Zhao H. Hou M. (2012). Acquired thermotolerance in plants. *Plant Cell Tiss. Org. Cult.* 111 265–276. 10.1007/s11240-012-0198-6

[B39] Su P. H. Li H. M. (2010). Stromal Hsp70 is important for protein translocation into pea and *Arabidopsis* chloroplasts. *Plant Cell* 22 1516–1531. 10.1105/tpc.109.071415 20484004 PMC2899880

[B40] Sun W. Van Montagu M. Verbruggen N. (2002). Small heat shock proteins and stress tolerance in plants. *Biochim. Biophys. Acta* 1577 1–9. 10.1016/S0167-4781(02)00417-7 12151089

[B41] Szklarczyk D. Gable A. L. Lyon D. Junge A. Wyder S. Huerta-Cepas J. (2019). STRING v11: protein-protein association networks with increased coverage, supporting functional discovery in genome-wide experimental datasets. *Nucleic Acids Res.* 47 D607–D613. 10.1093/nar/gky1131 30476243 PMC6323986

[B42] Tsvetkova N. M. Horvath I. Torok Z. Wolkers W. F. Balogi Z. Shigapova N. (2002). Small heat-shock proteins regulate membrane lipid polymorphism. *Proc. Natl. Acad. Sci. U.S.A.* 99 13504–13509. 10.1073/pnas.192468399 12368478 PMC129703

[B43] Tyanova S. Temu T. Sinitcyn P. Carlson A. Hein M. Y. Geiger T. (2016). The Perseus computational platform for comprehensive analysis of (prote)omics data. *Nat. Methods* 13 731–740. 10.1038/nmeth.3901 27348712

[B44] Van Aken O. Zhang B. Carrie C. Uggalla V. Paynter E. Giraud E. (2009). Defining the mitochondrial stress response in *Arabidopsis thaliana*. *Mol. Plant* 2 1310–1324. 10.1093/mp/ssp053 19995732

[B45] Vanderauwera S. Zimmermann P. Rombauts S. Vandenabeele S. Langebartels C. Gruissem W. (2005). Genome-wide analysis of hydrogen peroxide-regulated gene expression in *Arabidopsis* reveals a high light-induced transcriptional cluster involved in anthocyanin biosynthesis. *Plant Physiol.* 139 806–821. 10.1104/pp.105.065896 16183842 PMC1255997

[B46] Vaškebová L. Šamaj J. Ovecka M. (2018). Single-point ACT2 gene mutation in the *Arabidopsis* root hair mutant der1-3 affects overall actin organization, root growth and plant development. *Ann. Bot.* 122 889–901. 10.1093/aob/mcx180 29293922 PMC6215051

[B47] Wang M. Zou Z. Li Q. Xin H. Zhu X. Chen X. (2017). Heterologous expression of three *Camellia sinensis* small heat shock protein genes confers temperature stress tolerance in yeast and *Arabidopsis thaliana*. *Plant Cell Rep.* 36 1125–1135. 10.1007/s00299-017-2143-y 28455764

[B48] Wang W. Vinocur B. Shoseyov O. Altman A. (2004). Role of plant heat-shock proteins and molecular chaperones in the abiotic stress response. *Trends Plant Sci.* 9 244–252. 10.1016/j.tplants.2004.03.006 15130550

[B49] Waters E. R. (2013). The evolution, function, structure, and expression of the plant sHSPs. *J. Exp. Bot.* 64 391–403. 10.1093/jxb/ers355 23255280

[B50] Waters E. R. Vierling E. (2020). Plant small heat shock proteins – evolutionary and functional diversity. *New Phytol.* 227 24–37. 10.1111/nph.16536 32297991

[B51] Waters E. R. Lee G. J. Vierling E. (1996). Evolution, structure and function of the small heat shock proteins in plants. *J. Exp. Bot.* 47 325–338. 10.1093/jxb/47.3.325 12432039

[B52] Waters E. R. Nguyen S. L. Eskandar R. Behan J. Sanders-Reed Z. (2008). The recent evolution of a pseudogene: diversity and divergence of a mitochondria-localized small heat shock protein in *Arabidopsis thaliana*. *Genome* 51 177–186. 10.1139/G07-114 18356953

[B53] Waters M. T. Fray R. G. Pyke K. A. (2004). Stromule formation is dependent upon plastid size, plastid differentiation status and the density of plastids within the cell. *Plant J.* 39 655–667. 10.1111/j.1365-313X.2004.02164.x 15272881

[B54] Weigel D. Glazebrook J. (2002). *Arabidopsis: A Laboratory Manual.* New York, NY: Cold Spring Harbor Laboratory Press.

[B55] Zhang L. Li Y. Xing D. Gao C. (2009). Characterization of mitochondrial dynamics and subcellular localization of ROS reveal that HsfA2 alleviates oxidative damage caused by heat stress in *Arabidopsis*. *J. Exp. Bot.* 60 2073–2091. 10.1093/jxb/erp078 19342427

[B56] Zhang Y. Swart C. Alseekh S. Scossa F. Jiang L. Obata T. (2018). The extra-pathway interactome of the TCA cycle: expected and unexpected metabolic interactions. *Plant Physiol.* 177 966–979. 10.1104/pp.17.01687 29794018 PMC6052981

[B57] Zhao Y. Yu H. Zhou J. M. Smith S. M. Li J. (2020). Malate circulation: linking chloroplast metabolism to mitochondrial ROS. *Trends Plant Sci.* 25 446–454. 10.1016/j.tplants.2020.01.010 32304657

[B58] Zhong L. Zhou W. Wang H. Ding S. Lu Q. Wen X. (2013). Chloroplast small heat shock protein HSP21 interacts with plastid nucleoid protein pTAC5 and is essential for chloroplast development. *Plant Cell* 25 2925–2943. 10.1105/tpc.113.111229 23922206 PMC3784589

