# Supplementary material for: Mitochondrial Small Heat Shock Proteins Are Essential for Normal Growth of Arabidopsis thaliana
Source: Front Plant Sci. 2021 Feb 10;12:600426. doi: 10.3389/fpls.2021.600426 (PMC7902927; doi:10.3389/fpls.2021.600426)
Supplement: Supplementary file 1 [file Data_Sheet_1.zip › Supplementary Figures 1-6.PDF]

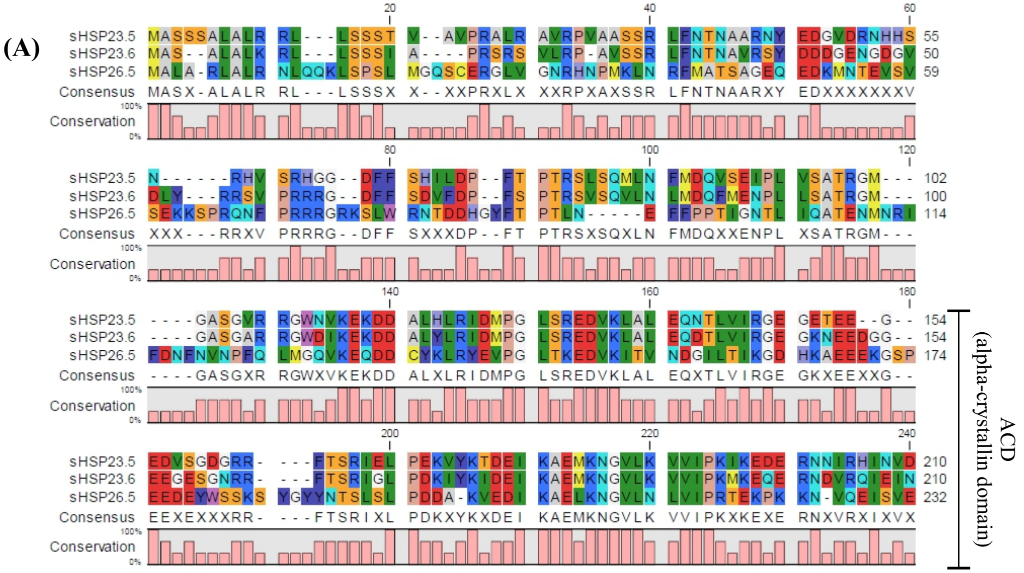

(B)

	sHSP26.5	sHSP23.5	sHSP23.6
**sHSP26.5**	100.00	32.99	33.51
**sHSP23.5**	32.99	100.00	68.29
**sHSP23.6**	33.51	68.29	100.00

Supplementary Figure S1. Sequence alignment of mitochondrial sHSP. (A) Protein sequences were aligned using the CLC Sequence Viewer 7.0.2 software. Colours were used to distinguish common residues and the consensus indicates amino acid present in at least two sequences. Pink bars represent the percentage of conservation of one position in the alignment. The mitocondrion transit peptide is located in 1-20 aa (sHSP23.5), 1-31 aa (sHSP23.6) and 1-42 aa (sHSP26.5), and the highly conserved ACD domain in 102-210 aa (sHSP23.5), 100-120 aa (sHSP23.6) and 114-232 aa (sHSP26.5) (Uniprot). (**B**) The percentage identity Matrix was created by Clustal2.1.

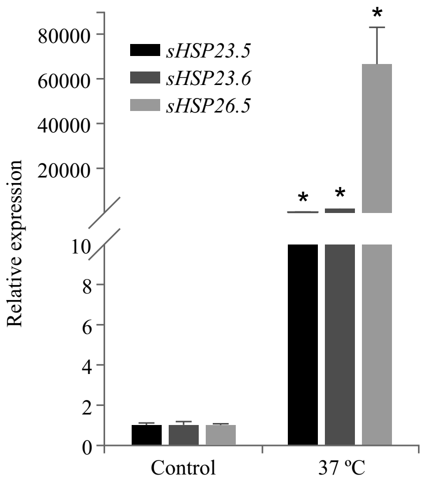

Supplementary Figure S2. Expression of *sHSP23.5*, *sHSP23.6*, and *sHSP26.5* in *A. thaliana* seedlings. RT-qPCR of *sHSP23.5*, *sHSP23.6*, and *sHSP26.5* in normal conditions and after heat stress. Plants were grown for 7 days at 22 °C under long day conditions and then exposed for 3 days to 37 °C. All expression values are normalized to *PP2A* as a reference. Relative expression of the transcripts was normalized to their respective expression levels at control conditions. Each data point represents the mean value ± SD of four biological replicates. Twenty plants were pooled for one replicate and the experiment were performed twice. Asterisks indicate significance by two-sided *t*-test with $*P < 0.01$.

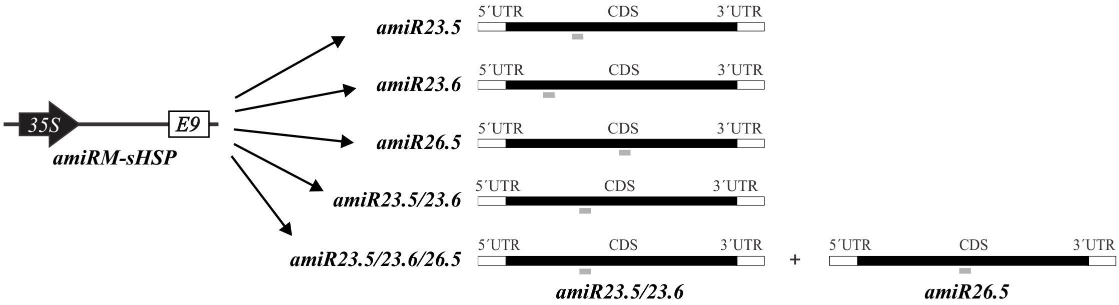

Supplementary Figure S3. Design of amicroRNA targeting *sHSP-M*. Site-directed mutagenesis and overlapping PCR were used to modify the MIR319a precursors and generate *amiR* for the sHSPs-M genes. In each construction, amiRs were designed to target a specific sequence (lines in grey) in the gene transcripts.

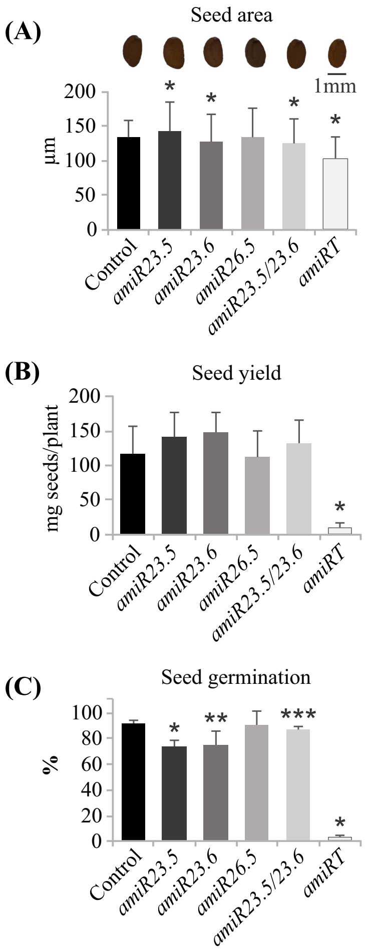

Supplementary Figure S4. Seed phenotype of knock-down and control plants. Seeds from senescent plants were collected, weighed and the areas were measured. For each *amiR*, seeds from 4 independent transgenic lines and 3 plants per line were analyzed. Measured seed areas (**A**) and seed yield (**B**). Data points represent the mean value ± SD of the 4 replicates. A minimum of 600 seed per transgenic line or control plant were measured for the seed area. For the germination assay, ca. 80 seeds of each *amiR* and control lines were surface sterilized and grown in petri dishes containing half-stregth Murashige and Skoog medium and solidified with 0.8 % (w/v) agar. Germination was calculated as the percentage of germinated seeds after 9 days (**C**). Data points represent the mean value ± SD of 4 independent transgenic lines evaluated in each *amiR* contruct. Asterisks indicate significance by two-sided *t*-test with $*P < 0.01$, $**P < 0.05$, $***P < 0.1$.

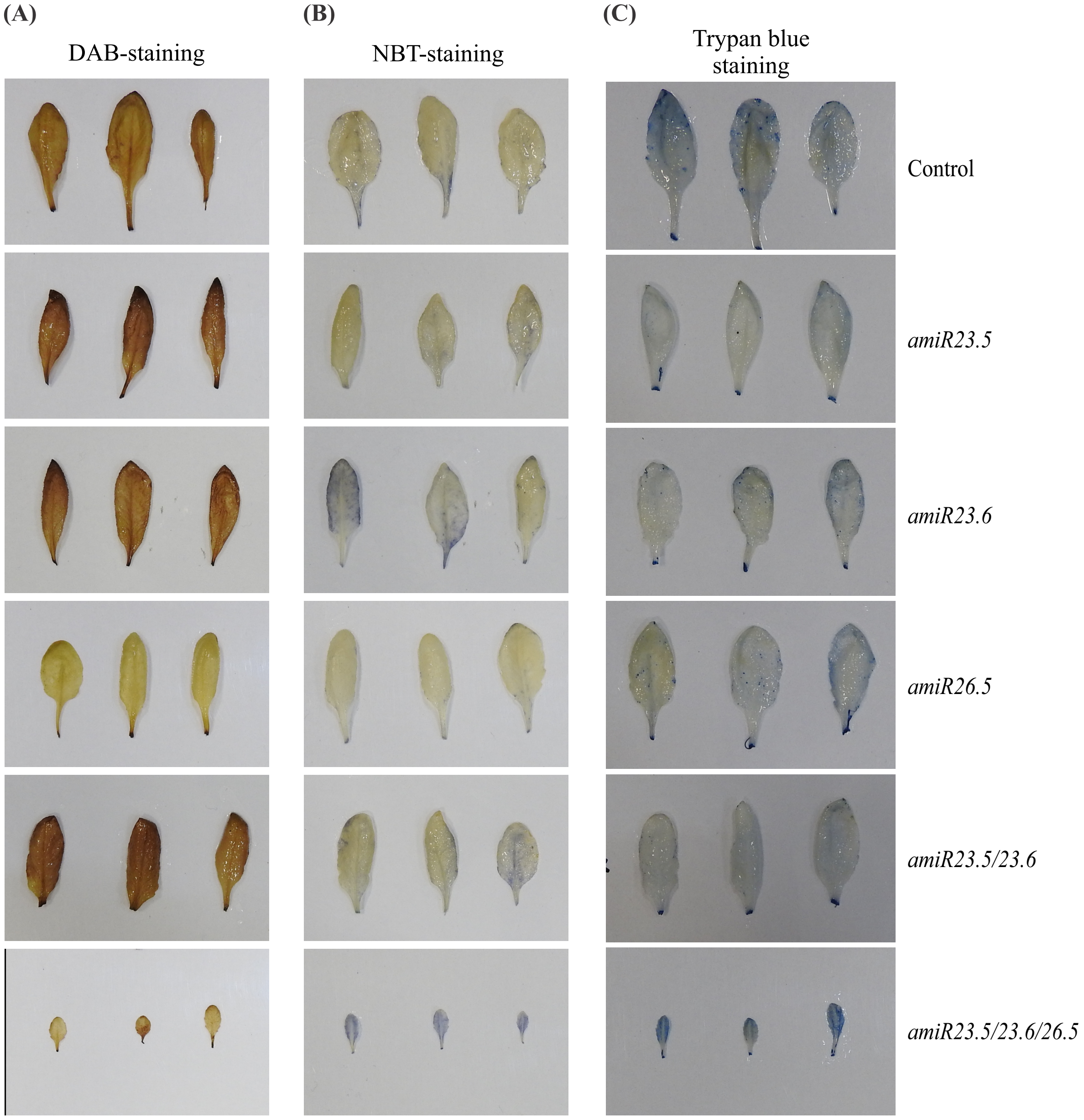

Supplementary Figure S5. Histochemical detection of reactive oxygen species and cell death in *amiR* plants. Detection of H_2O_2 by DAB-staining (**A**), $O_2^{\cdot -}$ by NBT-staining (**B**), and cell death by trypan blue-staining (**C**) was performed in leaves from 28 day-old plants under normal conditions. In (**C**) note the strong staining of the triple *amiR23.5/23.6/26.5* leaves. Four independent lines of each *amiR* construct and 8 biological replicates per control and *amiR* line were analyzed. Three representative leaves are shown.

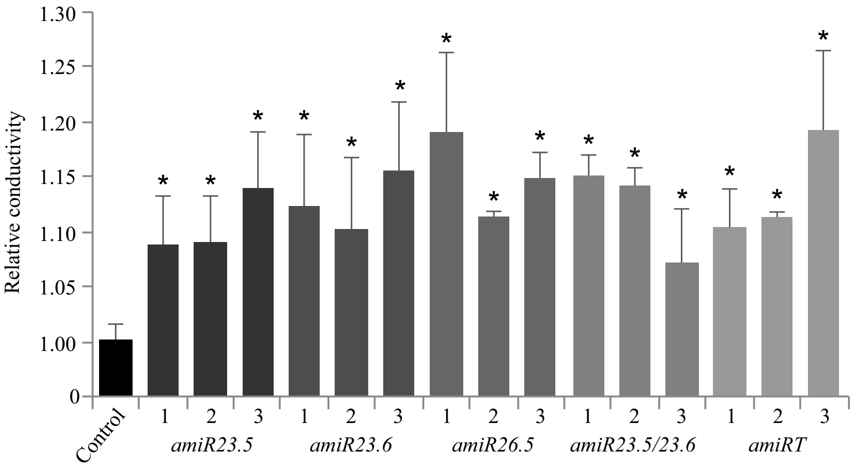

Supplementary Figure S6. Electrolyte leakage in *amiR* and control plants. Determinations were performed on discs from leaves of 28 day-old plants. Electrolyte leakage was estimated by measuring the conductivity with a conductance meter (Twin Compact Meter-Horiba, Northampton, UK). Three independent transgenic lines were measured for each *amiR*. Results are presented as the conductivity relative to the control plants values. Data points represent the mean value ± SD of three biological replicates. Asterisks indicate significance by two-sided *t*-test with $P<0.05$.
